# Transcription factors in the pathogenesis of pulmonary arterial hypertension—Current knowledge and therapeutic potential

**DOI:** 10.3389/fcvm.2022.1036096

**Published:** 2023-01-06

**Authors:** Jakob Körbelin, Julius Klein, Christiane Matuszcak, Johannes Runge, Lars Harbaum, Hans Klose, Jan K. Hennigs

**Affiliations:** ^1^ENDomics Lab, Department of Medicine, Center of Oncology, University Medical Center Hamburg-Eppendorf, Hamburg, Germany; ^2^Division of Pneumology and Center for Pulmonary Arterial Hypertension Hamburg, University Medical Center Hamburg-Eppendorf, Hamburg, Germany

**Keywords:** epigenetics, epigenetics (chromatin remodeling), transcriptomics, targeted therapy, reverse remodeling, pathogenesis, pulmonary hypertension (PAH)

## Abstract

Pulmonary arterial hypertension (PAH) is a disease characterized by elevated pulmonary vascular resistance and pulmonary artery pressure. Mortality remains high in severe cases despite significant advances in management and pharmacotherapy. Since currently approved PAH therapies are unable to significantly reverse pathological vessel remodeling, novel disease-modifying, targeted therapeutics are needed. Pathogenetically, PAH is characterized by vessel wall cell dysfunction with consecutive remodeling of the pulmonary vasculature and the right heart. Transcription factors (TFs) regulate the process of transcribing DNA into RNA and, in the pulmonary circulation, control the response of pulmonary vascular cells to macro- and microenvironmental stimuli. Often, TFs form complex protein interaction networks with other TFs or co-factors to allow for fine-tuning of gene expression. Therefore, identification of the underlying molecular mechanisms of TF (dys-)function is essential to develop tailored modulation strategies in PAH. This current review provides a compendium-style overview of TFs and TF complexes associated with PAH pathogenesis and highlights their potential as targets for vasculoregenerative or reverse remodeling therapies.

## Introduction

Pulmonary arterial (PA) hypertension (PAH), whether idiopathic (IPAH), hereditary (HPAH), or associated with other conditions (APAH), is a rare, serious and progressive pulmonary vascular disease. Despite improvements in the management of PAH, overall 5-year mortality remains around 30% (1).

PAH is characterized by elevated resistance and pressure in precapillary pulmonary vessels leading to right heart failure, if untreated (2, 3). Pathophysiologically, PAH is characterized by an initial loss of small pulmonary microvessels via endothelial cell (EC) apoptosis in combination with neointima formation through the uncontrolled growth of smooth muscle cell (SMC)-like cells, adventitial fibroblasts (AF), pericytes and mesenchymally transdifferentiated endothelial cells (endothelial-mesenchymal transition, EndMT) (4–6). Although the origin of hyperproliferative neointimal cells in PAH is still not fully understood, recent lineage-tracing studies suggest that the neointima mainly consists of propagating SMC, while EndMT can be detected in a smaller fraction of pathologically remodeled lung vessels (7). Upon persistent vascular inflammation, PAECs also undergo a phenotypic switch from initially increased propensity to apoptosis toward a more apoptosis-resistant and hyperproliferative state thereby further contributing to intraluminal PA obstruction (4, 8).

Currently available pharmacological options in PAH comprise vasodilatory drugs with selectivity for the pulmonary vasculature that attenuate disease progression; namely endothelin receptor antagonists (ERA: bosentan, ambrisentan, and macitentan), phosphodiesterase 5 inhibitors (PDE5i: sildenafil, tadalafil), or soluble guanylate cyclase (sGC: riociguat) stimulator in addition to prostanoids/prostacyclin receptor agonist (epoprostenol, iloprost, treprostinil, selexipag) (9, 10). However, all of the currently available drugs fail to meaningfully reverse PAH-associated structural remodeling of pulmonary blood vessels and lung transplantation remains the only cure. Therefore, novel therapeutic approaches are needed to attenuate PAH progression but also reverse prevalent structural remodeling of the pulmonary vasculature.

In this light, disease-modifying drugs have been an important research focus in PAH over the last few years. Bone morphogenic protein receptor type II (BMPR2) has evolved as a promising molecular target (11, 12). BMPR2 is a transmembrane serine/threonine receptor kinase and a member of the transforming growth factor (TGF)-β superfamily and is a pivotal player in differentiation, inflammation, apoptosis, and proliferation pathways of the pulmonary vasculature (4, 13–15). Pathogenic variants in the *BMPR2* gene account for approx. 75% of HPAH cases and for ∼20% of IPAH cases (16, 17). In addition to germline mutations, BMPR2 expression and BMPR2 signal transduction is universally impaired in all PAH forms, including APAH (16, 18–20) and other precapillary PH forms such as chronic thromboembolic PH and interstitial lung disease associated PH (21, 22) by a plethora of pathological mechanisms [reviewed in (23)]. Pharmacological strategies to re-activate or re-balance BMPR2 signaling in the pulmonary vasculature have been able to restore PA endothelial function, suppress PASMC proliferation and successfully treat PH in experimental models (24–28) and early clinical trials (29–31).

Downstream of BMPR2, non-canonical transcription factors (TFs) can be pharmacologically harnessed to reverse experimental PH (28, 32–35) and repair prevalent DNA damage in PAEC from PAH patients harboring BMPR2 mutations (28) uncovering an additional BMPR2-dependent disease-modifying approach.

This review, therefore, summarizes the current knowledge regarding the role of TFs in PAH pathogenesis and explores their therapeutic potential as disease modifiers in PAH.

## Transcription factors: Molecular basics

TFs are key cellular components that—as molecular switches—control gene expression: TFs are DNA-binding proteins that relay external and internal cellular stimuli to a molecular function enabling gene transcription (36). These processes require modification in chromatin structure by chemical modification of DNA and histones as well as other ribonucleoproteins. Therefore, TFs are part of a finely tuned interaction network with chromatin remodeling or histone-modifying proteins to regulate gene transcription (37). TFs bind to highly specific regulatory DNA elements, so called “motifs,” within promoter or enhancer regions of their target genes to either activate or repress transcription (38, 39). TFs can regulate transcription either by recruiting chromatin remodeling proteins to induce conformational changes of chromatin to provide DNA accessibility or by directly binding to promotors and enhancers to facilitate the recruitment of additional components of the transcriptional machinery for transcription initiation (37). In this regard, TFs have much higher (> 1,000-fold) affinity to their specific DNA-binding sites within a target gene (= TF-binding site, TFBS) than to surrounding, non-specific DNA sequences (40). These TFBS (or “motifs”) are usually found as DNA repeats in cis-regulatory and non-coding DNA elements (see above) (41). As TFs are pivotal to integrating a plethora of cellular processes, TF dysfunction, e.g., through mutations, or (epigenetic) inactivation, contributes to the pathogenesis of numerous diseases (41–45).

## Transcription factors: Key regulators in PAH pathogenesis

**FIGURE 1:**
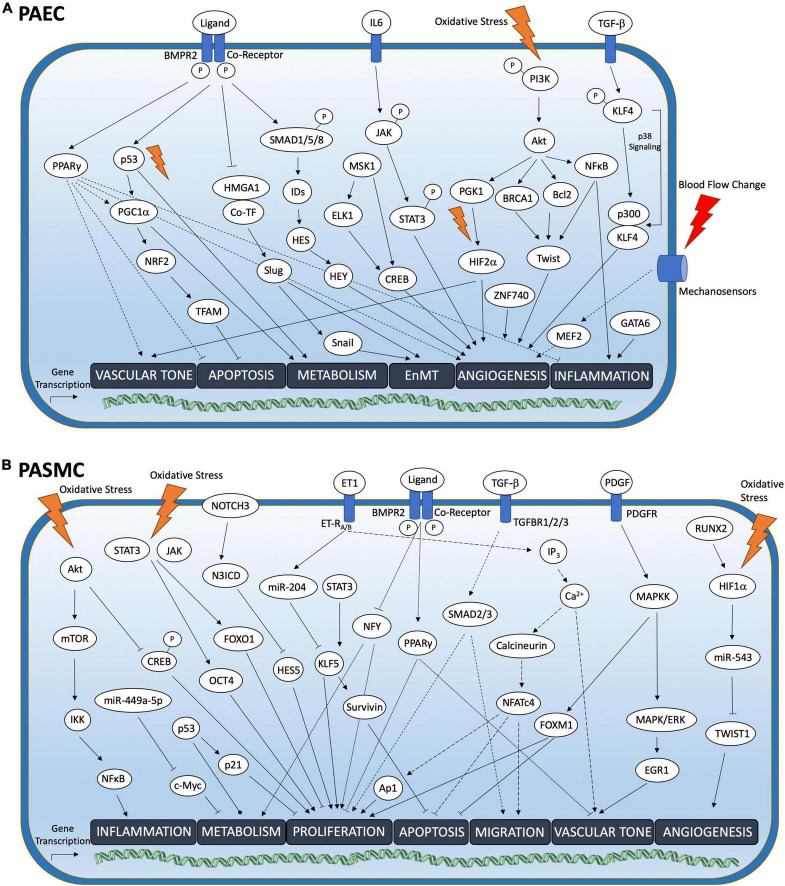
Transcription factor pathways in PAEC and PASMC associated with PAH. Overview of pathogenetically relevant transcription factor (TF) pathways in **(A)** pulmonary arterial endothelial cells (PAEC) and **(B)** pulmonary arterial smooth muscle cells (PASMC) upon activation by cell membrane anchored receptor signaling or cellular stress events. Depending on the pulmonary vascular cell type TF activation mediated gene transcription elicits cell-type specific downstream responses. Blue box represents cell membrane. Ca^2+^, Calcium; IP_3_, Inositol 1,4,5-trisphosphate; ET-R_A/B_, Endothelin Receptor Type A and B; TGFBR1/2/3, TGF-ß receptor 1/2/3; −p, Phosphorylation.

In the pulmonary vasculature, TFs regulate crucial cellular functions such as proliferation, differentiation, inflammation, cell death, repair, and regenerative programs (39, 45). In PAH, TFs are responsible for altered expression of multiple disease-related genes thereby contributing to defective cellular homeostasis and vascular remodeling (46, 47). Members from eight out of ten TF superclasses (48) are crucially involved in PAH pathophysiology. In this section, we provide a short compendium of the most relevant TFs of each superclass with relevance to PAH (please also see Table 1 and Figures 1A,B).

**TABLE 1:** Transcription factors in PAH.

TF superclass (S1–6)	TF name	Cell type	Expression and function in PAH	References
S1: Basic domains group	CREB	PASMC	Expression: ↓ Function: proliferation↓, migration↓, hypertrophy↓, dedifferentiation↓ and ECM production↓	(49)
	TWIST1	PAEC	Expression: ↑ Function: EndMT↑, vascular remodeling↑	(56)
	MYC	PASMC	Expression: ↑ Function: regulates mitochondrial and metabolic function (in PAH: under hypoxia-induced phenotype transformation proliferation↑ and hypoxia-induced mitochondrial dysfunction↑)	(55)
	HIF1A	PAEC, PASMC	Expression: ↑ Function: metabolic shift↑ (anaerobic glycolysis), angiogenesis↑, proliferation↑, inflammation↑, apoptosis↓	(150, 155, 156, 167–169)
	HIF2A	PAEC, LVEC	Expression: ↑ Function: EndMT↑ via SNAI1/2↑, vascular remodeling↑, occlusive lesions↑, influences vascular resistance	(172)
	HES5	PASMC	Expression: ↑ Function: proliferation effect of NOTCH3↑, gene expression shift into undifferentiated phenotype↑	(51)
	AP1	PASMC	Expression: c-fos↑, c-jun ↑ Function: involved in proliferative response via ET1	(147)
S2: Zinc-coordination DNA-binding domains	PPARγ	PAEC	Expression: ↓ Function: cell cycle progression↑, cell survival↑, apoptosis↓	(32, 46, 47, 60, 63, 66, 70)
		PASMC	Expression: ↓ Function: vessel remodeling↓, proliferation↓, mitochondrial integrity↑, apoptosis↑	
	SNAI2	PAEC	Expression: ↑ Function: EndMT↑ via HMGA1 after BMPR2↓	(57)
	EGR1	PASMC, PAAF	Expression: ↑ Function: vessel remodeling↑, medial hypertrophy↑	(73, 75, 233)
	ZNF740	PAEC	Expression: ↑ Function: proliferation↑, angiogenesis↑	(76)
	KLF2	PAEC	Expression: ↓ Function: proliferation↓, apoptosis↓, inflammation↓, vasodilation↑	(80–82)
	KLF4	PAEC	Expression: ↓ Function: vessel protection↑, regulation of vasodilation, inflammation↓, coagulation↓, and oxidative stress, chromatin accessibility for vasculoprotective genes↑	(83, 84, 234)
	KLF5	PASMC	Expression: ↑ Function: proliferation↑, apoptosis↓	(85)
	PPARGC1A (PGC1A)	PBMC	Expression: ↑ under hypoxia Function: Regulates total antioxidant status via CYTC and SOD, inflammation by activating CYTC	(20, 87, 88)
		PASMC	Expression: ↓ Function: mitochondrial integrity ↑, maintains proliferation-apoptosis rheostat	(88)
		PAEC	Expression: Under BMPR2-loss and normoxia↑, hypoxia↓ hypoxia-reoxygenation↓↓ Function: Promotes mitochondrial health and integrity upon oxidative stress via NRF2-TFAM cascade	(20)
	GATA6	PAEC	Expression: ↓ Function: transcription regulator of genes controlling vascular tone, inflammation and vascular remodeling	(89)
S3: Helix-turn-helix domains	FOXO1	PASMC	Expression: ↓ Function: proliferation↓	(93)
	FOXM1	PASMC	Expression: ↑ Function: proliferation↑, DNA-repair↑, resistance to apoptosis↑	(90, 92, 235)
	ELK1	PAEC	Expression: ↑ Function: proliferation↑	(95)
	MSX1	Lymphocytes	Expression: ↑ (under BMPR2-loss) Function: capillary regression↑	(96)
	OCT4	PASMC	Expression: ↑ (PSG1 + 5: ↓) Function: proliferation↑ under hypoxia	(98)
S4: Other all-alpha-helical DNA binding domains	SOX17	PAEC	Expression: ↓ Function: Regulates Notch-signaling in pulmonary EC development, PA remodeling↓, SNPs in SOX17 enhancer associated with impaired survival in PAH?	(101, 102, 105)
	TFAM	PAEC	Expression: under BMPR2-loss: normoxia↑, reoxygenation↓ Function: modulates inflammatory response, mtDNA integrity↑, EC survival↑	(20)
	NFY	PASMC	Expression: ↑ Function: regulates genes for proliferation, glycolysis, apoptosis-resistant phenotype↑	(106)
S5: alpha-Helices exposed by beta-structures	MEF-2	PAEC	Activity: ↓ Function: regulates expression of transcriptional targets involved in vessel homeostasis	(107)
S6: Immunoglobulin fold	NFAT	PASMC	Expression: ↑ Function: proliferation↑, migration↑, apoptosis-resistant phenotype↑, Warburg-phenotype↑	(58, 109, 110, 236)
	RUNX1	EPC	Expression: ↓ Function: EHT↑	(111)
	RUNX2	PASMC	Expression: ↑ Function: proliferation↑, vascular remodeling↑, calcification in PA lesions↑, resistance to apoptosis↑, transdifferentiation into osteoblast-like cells↑	(116)
	p53	PASMC	Expression: under hypoxia↓ Function: aerobic glycolysis↓, mitochondrial respiration↑, proliferation↓	(20, 128)
		PAEC	Expression: under BMPR2-loss and normoxia↑, hypoxia↓, hypoxia-reoxygenation↓ Function: p53↓: mtDNA deletion↑, apoptosis↑ p53↑: mitochondrial membrane potential↑, ATP production↑, glycolysis↑, production of cytokines↑	
	NF-KB	PAEC, PASMC	Expression: ↑ Function: inflammation by activation of macrophages, lymphocytes and endothelial cells↑, vascular remodeling↑, EndMT↑	(131)
	TBX4	−	TBX4 mutation associated with childhood-PAH and PAH with lung parenchymal maldevelopment	(136)
	STAT3	PASMC	Expression: ↑ Function: proliferation↑, resistance to apoptosis↑	(137, 184)
	STAT1	PAEC	Expression: ↑ Function: proliferation↑, migration↑, inflammation↑	(138)
S7: beta-Hairpin exposed by an alpha/beta-scaffold	SMAD3	PASMC	Expression: ↓ Function: proliferation↓, migration↓, vascular remodeling↓	(139)
		PAEC	Expression: ↓ (↑ under HERV-K dUTPase stimulation) Function: proliferation↓, migration↓, EndMT↑	(132)
S8: beta-Sheet binding to DNA	HMGA1	PAEC	Expression: ↑ Function: EndMT into SM-like phenotype↑ (with SNAI2)	(57, 139)

TFs by superclass as determined by comparison with the Human Transcription Factor Database (Animal TFDB 3.0; 232) and TFclass (48).

### TF superclass 1

*TFs belonging to the basic domains group superclass (S1) bind DNA through a basic region which becomes folded in an alpha-helically manner if added to DNA* (48). *At least six members of this superclass contribute to PAH pathogenesis.*

#### CREB

*Cyclic adenosine monophosphate (cAMP) response element binding protein* (CREB) functions as an anti-proliferative TF in healthy PASMC. In PAH and associated oxidative stress with excessive production of reactive oxygen species (ROS) like H_2_O_2_, CREB is downregulated leading to enhanced proliferation of PASMC (49).

#### HES5

The *hes family bHLH transcription factor 5* (HES5) binds to the Notch-receptor and promotes proliferative signals (50). In PASMCs, HES5 inactivation reverses the proliferative effect of NOTCH3 and induces a shift in gene expression toward a more differentiated phenotype (51).

#### MYC

MicroRNAs (miR/miRNA) regulate numerous disease pathways in the pulmonary vasculature and have been linked with PAH development (52–54). In this context, Zhang et al. showed that miR-449a-5p, which is downregulated in PAH, represses the activity of the TF *MYC proto-oncogene* (MYC). Lack of MYC repression in PAH PASMC is associated with mitochondrial and metabolic dysfunction as well as phenotype transformation (55).

#### TWIST1

Expression of *Twist-related protein 1* (TWIST1) is increased in the lungs of PAH patients and TWIST1 has been shown to mediate EndMT thereby contributing to pathological vascular remodeling in PAECs (56, 57).

### TF superclass 2

*The TF superclass 2 contains TFs with Zinc-coordination DNA-binding domains. Such zinc fingers, consisting of a repetitive pattern of cysteine and histidine residue, represent the most frequent DNA-binding motifs found in eukaryotic TFs* (48). *The frequency of zinc fingers among DNA-binding motifs is also represented by the many members of the TF superclass 2 that play a role in PAH.*

#### PPARG

*Peroxisome proliferator-activated receptor gamma* (PPARγ) is a member of the nuclear hormone receptor superfamily of ligand-activated TFs. It is pivotal for the regulation of mulitple central processes in pulmonary vascular cells (47, 58–61). PPARγ, which is ubiquitously expressed, represents probably the best-studied TF in pulmonary hypertension. Norbert Voelkel and his group were first to demonstrate that PPARγ is downregulated in lungs from PAH patients and in PAH-associated vascular lesions (62). PPARγ dysfunction in PAEC or PASMC facilitates the hyperproliferative vascular phenotype typical for PAH (47, 63).

In PASMC, a downregulation of PPARγ by short interfering RNA leads to increased proliferation, decreased mitochondrial mass and increased mitochondrial ROS generation (47, 63), which is in part mediated by decreased levels of TFAM, GRP75, and MFN2 (47) and by NF-kB dependent NOX4 upregulation (64). In contrast, pharmacological PPARγ activation is sufficient to reverse experimental PH (58, 65).

In this regard, Hansmann et al. showed that PPARγ-mediated anti-proliferative BMP-2 signaling in PASMC and that loss of PPARγ function in PASMC was associated with the spontaneous onset of experimental PH. PPARγ agonists were able to restore anti-proliferative signaling in wildtype and BMPR2-mutant PASMC, suggesting early on that activation of PPARγ signaling may reverse PAH (66). Mechanistically, in PASMC this is mediated by BMP2-dependent upregulation of a protective autocrine PPARγ—Apolipoprotein E (ApoE)—Low density lipoprotein receptor-related protein 1 (LRP1) axis (66) and inhibition of TGF1-mediated SMAD3/4 and STAT3-FOXO1 signaling (see below) (67). In these studies, Chakraborty et al. used Cre-constructs driven by the Tagln/Sm22-promoter to delete PPARγ in SMC instead of more SMC-specific promoters such as Myh11 (68). The Tagln/Sm22 promoter has been shown to also be active in cardiomyocytes and non-muscle tissues such as myeloid cells and platelets [reviewed in: (68)]. Therefore, future studies need to evaluate to what extent PPARγ’s protective function to reverse experimental PH relates to rehabilitation of SMC-specific signaling or also includes effects on additional cell types such as cardiomyocytes as suggested by a recent study of the same group (32).

In PAECs, Vattulainen-Collanus et al. suspected that a lack of PPARγ could result in increased expression of E2F1, which is associated with a dysregulated Wnt pathway and disturbed angiogenesis and migration (69). PPARγ may also play a role in PAEC’s response to DNA damage (70). In cellular studies, depletion of PPARγ was sufficient to promote the development of a PH phenotype by upregulation of cell cycle- and angiogenesis-related genes (71). In an EC-specific PPARγ knockout mouse model (using the Tie2 promoter), experimental PAH developed spontaneously (63). Additional information on the beneficial effects of PPARγ on the pulmonary vasculature can be found further down in the section on PPARγ TF complexes.

#### SNAI2

*Snail family transcriptional repressor 2* (SNAI2), also known as Slug, a highly conserved zinc finger TF, has been implicated in epithelial-mesenchymal transition (EMT) and EndMT (72). In PAEC, loss of BMPR2 leads to increased expression of High-mobility group protein 1 (HMGA1) and Slug, which is associated with upregulation of SMC markers and EndMT (57).

#### EGR1

Expression of *early growth response protein 1* (EGR1) is increased in plexiform lesions of PAH (73, 74), is triggered by tissue damage and is associated with pathological remodeling of the lung vessel wall (75). Interestingly, EGR-1 is negatively regulated by PPARγ agonists (75).

#### ZNF740

*Zinc finger proteins* (ZNFs) bind classically to DNA, RNA, proteins, and other small molecules and are highly conserved in their binding specificity of a particular protein. Yu et al. identified a novel signaling pathway involved in proliferation and angiogenesis of PAECs and in vascular remodeling *in vitro*. This new signaling axis consists of ZNF740, GDF11, TGF-β-receptor I, and SMAD, which is also involved in the imbalance of pulmonary vascular homeostasis in PAH (76).

#### KLF2

*Krüppel-like Factor 2* (KLF2) is a vasculoprotective factor expressed in endothelial cells that is activated by laminar shear stress and is pivotal for normal lung vessel formation (77). Heterozygous germline missense mutations in KLF2 have recently been associated with HPAH (Table 2) (78–80) and KLF2 mRNA expression is strongly downregulated in lungs from rodents and humans with PAH (80, 81). Loss of KLF2 impairs NO synthesis and thereby contributes to the severity of hypoxia-induced PH in Apelin-deficient mice (82). In contrast, adenoviral transduction mediates anti-inflammatory, anti-apoptotic, and anti-proliferative effects in PAEC under nutrient stress (80). Additionally, miRNA isolated from exosomes derived from KLF2-overexpressing PAEC can be therapeutically harnessed to attenuate experimental PH in the Sugen/hypoxia mouse model (80).

**TABLE 2:** Genetic variants in transcription factors associated with PAH pathogenesis.

Gene symbol	Identifier	Location	Mode of inheritance	Gene-disease validity assertion
SMAD9	HGNC:6774	Chr 13 (36844831.36920854)	Autosomal dominant	Definitive (ClinGen) high evidence (Genomics England) high evidence (BRIDGE consortium)
TBX4	HGNC:11603	Chr 17 (61452422.61485110)	Autosomal dominant	Definitive (ClinGen) high evidence (Genomics England) n/a (BRIDGE consortium)
SOX17	HGNC:18122	Chr 8 (54457935.54460892)	Autosomal dominant	In scope (ClinGen) high evidence (Genomics England) n/a (BRIDGE consortium)
SMAD1	HGNC:6767	Chr 4 (145480770.145559176)	(Pseudo-) autosomal dominant	In scope (ClinGen) low evidence (Genomics England) high evidence (BRIDGE consortium)
SMAD4	HGNC:6770	Chr 18 (51030213.51085042)	(Pseudo-) autosomal dominant	In scope (ClinGen) low evidence (Genomics England) high evidence (BRIDGE consortium)
KLF2	HGNC:6347	Chr 19 (16324826.16328685)	Autosomal dominant	In scope (ClinGen) n/a (Genomics England) n/a (BRIDGE consortium)

TFs are sorted by evidence level of variant-disease association as determined by three consortia (ClinGen Genomics England and BRIDGE consortium). Chr, Chromosome; HGNC, HUGO Gene Nomenclature Committee.

#### KLF4

*Krüppel-like Factor 4* (KLF4), a protective PAEC maintenance factor, is inactivated by posttranslational modification upon nitrosative stress, thereby disabling its protective function in the pulmonary vasculature (83). Recently, KLF4 was identified as an interaction partner of the SWI/SNF complex to increase accessibility of enhancer sites which regulate genes essential for endothelial homeostasis under laminar shear stress (84).

#### KLF5

*Krüppel-like Factor 5* (KLF5) has been linked with an apoptosis-resistant and proliferative phenotype in PASMCs (85), as an upstream regulator of HIF1 in PASMC (86). In addition, KLF5 and HIF1 might form a TF complex with yet unknown function (86).

#### PGC1A

*PPAR*γ *coactivator-1*α (PGC1A/PPARGC1A), which normally regulates oxidative metabolism and mitochondrial biogenesis, was found to regulate inflammation in blood cells of IPAH patients by activating cytochrome complex (CYTC) under hypoxia (87, 88). In PASMC, PGC1A regulates the expression of the Mitofusin-2 gene MFN2 to maintain mitochondrial integrity. PAH PASMC lacking PGC1A and MFN2 show heightened mitochondrial fragmentation associated with increased PASMC proliferation (88). In PAEC, PGC1A promotes EC survival and sustains mitochondrial membrane potential upon oxidative stress downstream of a non-canonical BMPR2-p53 axis (20).

#### GATA6

*GATA sequence binding protein 6* (GATA6), a member of the ZNF TF family, is upregulated in inactive vasculature and downregulated during vascular injury (89). In PAECs, GATA6 directly regulates ET1 receptor type A (ETA), a gene for controlling vascular tone, as well as pro-inflammatory genes like 5-lipoxygenase-activating protein PAI-1, which is involved in vascular remodeling and increased vascular muscularization (89).

### TF superclass 3

*The helix-turn-helix superclass (S3) of TFs comprises a DNA-recognition helix that fits into the major DNA groove. Some important TFs regarding their relevance to PAH belong to this superclass.*

#### FOXO1 and FOXM1

*Forkhead box proteins O1* (FOXO1) and *M1* (FOXM1) have opposing roles in PAH pathogenesis. While FOXM1 is overexpressed in PASMC of PAH patients and promotes hypoxia-induced proliferation as well as resistance against apoptosis and DNA repair (90–92), FOXO1, which integrates multiple vasculoprotective pathways, shows reduced expression and/or is inactivated in PAH PASMC (93).

#### ELK1

*ETS Like-1 protein* (ELK1) is a member of the E-twenty-six (Ets) ternary complex family of TFs known to stimulate the expression of immediate early response genes involved in cellular proliferation and apoptosis (94). Phosphorylation of Elk-1 in concert with p38-mitogen-activated protein kinase (MAPK) induces PAEC proliferation (95).

#### MSX1

*Msh homeobox 1* (MSX1) is upregulated in lymphocytes of IPAH patients and EC of BMPR2-deficicent mice. Lack of BMPR2-mediated suppression derepressed MSX1 expression which correlates with upregulation of MSX1 target genes in IPAH (96).

#### OCT4

*The octamer-binding TF 4* (OCT4) is a marker for undifferentiated cells, highly expressed in human embryonic stem cells. Even though OCT4 is frequently silenced in differentiated somatic cells (97), Firth et al. detected weak expression of OCT4 isoforms A and/or B mRNA and strong expression of OCT4 pseudogene (PSG) 1 and 5 mRNA in PASMC from healthy controls. In PASMCs under hypoxia or isolated from IPAH patients, mRNA expression of OCT4A/B is upregulated, whereas OCT4 PSG 1 and 5 are downregulated (98). OCT4A/B upregulation in IPAH PASMC might be mediated by HIF2α, which has been shown to directly bind to the OCT4 promoter (99), and is a key regulator of the pro-proliferative response in PAAF (100). This is in line with a study by Bertero et al. showing that HIF2α-dependent OCT4 activation promotes early vascular stiffening as a central pathological event in PAH via induction of microRNA 130/301 (53). Therefore, hypoxia-associated OCT4 upregulation might also contribute to a hyperproliferative, dedifferentiated PASMC phenotype in IPAH.

### TF superclass 4

*TF superclass 4 comprises transcription factors with alpha-helical DNA-binding domains. At least three members of this superclass have important functions in PAH pathogenesis.*

#### SOX17

*SRY-related HMG-box (SOX) 17* is an endothelial-specific TF pivotal for cardiac and pulmonary development by integrating and regulating VEGF, WNT and NOTCH signaling [reviewed in (101)]. Activation of SOX17 represses PA remodeling in the monocrotaline PH model (102). Using genome-wide association studies in PAH, rare pathogenic variants within the coding region of SOX17 and SNPs in an enhancer region have been associated with PAH (103–105).

#### TFAM

*Transcription Factor A, Mitochondrial* (TFAM) is a crucial modulator of the inflammatory response to oxidative stress and maintains mitochondrial DNA integrity and cell survival in PAEC under oxidant stress downstream of the non-canonical BMPR2-p53 signaling axis (20).

#### NFY

*Nuclear factor Y* (NFY) is epigenetically activated in PASMC isolated from PAH patients to induce pro-proliferative and glycolysis genes to facilitate the cancer-like hyperproliferative and glycolytic-switch phenotype of PAH PASMC (106).

### TF superclass 5

*Members of the alpha-helices exposed by beta-structures (S5), as the name suggests, possess alpha helices exposed by a scaffold of beta-strands* (48). *To our knowledge, there is a single TF of this group with a well-established role in PAH.*

#### MEF2

Transcriptional activity of *myocyte enhancer factor 2* (MEF2) is inhibited in PAEC isolated from PAH patients by nuclear accumulation of histone deacetylases 4 and 5. Thereby, expression of vasculoprotective factors miR-424, miR-503, connexins 37 and 40 as well as KLF2 and 4 is impaired contributing to PAH pathogenesis (107).

### TF superclass 6

*The Immunoglobulin fold TF superclass (S6) comprises TFs that are characterized by a beta-core structure that induces a DNA contact. Many TFs of this group play a role in the context of PAH.*

#### NFAT

*Nuclear factor of activated T cells* (NFAT), discovered approx. three decades ago (108), is increased in PAH and regulates PASMC calcium homeostasis in conjunction with calcineurin (CaN) as interaction partner (109). Increased CaN/NFAT promotes PASMC proliferation, survival and migration in chronic hypoxia and MCT-induced PAH (109). In addition, NFAT is upregulated by DNA-damage mediated PARP-1 activation facilitating pulmonary vascular remodeling which was reversible by PARP-1 inhibitors (110).

#### RUNX1

Liang et al. reported that bone-marrow derived endothelial progenitor cells (EPC) undergo endothelial-to-hematopoietic transition (EHT) to promote pulmonary arterial hypertension. Inhibition of the critical hematopoietic transcription factor *Runt-related transcription factor 1* (RUNX1), also known as acute myeloid leukemia 1 protein (AML1), blocked EHT *in vivo*, and attenuated progression of experimental PH by preventing bone-marrow egression of EPC (111). In addition, RUNX1 mediates expression of neutrophil elastase in PASMC contributing to ECM remodeling in the pulmonary vasculature (112).

#### RUNX2

*RUNX family transcription factor 2* (RUNX2) promotes vascular remodeling and stiffening in vascular disease (113–115). RUNX2 activation promotes vascular calcification. Excessive proliferation of PASMCs in PAH is sustained over time by the loss of miR-204-mediated upregulation of RUNX2 contributing to the development of proliferative and calcified PA lesions (116).

#### TP53

*Tumor protein p53* (p53), the *Guardian of the Genome* (117), is a crucial TF highly conserved in multicellular vertebrates, where it functions as a tumor suppressor by maintaining genome integrity and stability (118). In general, p53 controls many central cellular functions such as cell cycle, DNA repair, apoptosis as well as inflammatory and metabolic homeostasis via its numerous (direct) target genes (119, 120). In the vasculature, depending on the context, p53 exerts both, detrimental (121–123) and regenerative effects (124, 125). In the pulmonary vasculature, p53 fulfills protective functions: Mizuno et al. demonstrated that mice with global p53 knockout developed more severe PH upon chronic hypoxia (126). This is in concert with data showing that pharmacological inhibition of p53 transcriptional activity by Pifithrin-α was sufficient to spontaneously induce PH in rats and to aggravate MCT-induced PH (127). In addition, Wakasugi et al. found that reduced p53 expression in PASMC led to increased aerobic glycolysis and downregulation of mitochondrial respiration thereby contributing to the cancer-like hyper-proliferative “Warburg phenotype” found in PASMC isolated from PAH patients. PASMC-specific p53-knockout, however, did not aggravate hypoxia-induced PH (128). Activation of p53 in PASMC by Nutlin-3, on the other hand, prevented and reversed experimental PH mice (129). In PAEC, p53 is a non-canonical effector downstream of BMPR2 (20, 28). Under oxidative stress, BMPR2-defective PAEC are unable to stabilize and transcriptionally activate p53 and p53-dependent TFs PGC1A, nuclear factor erythroid 2-related factor 2 (NRF2), and mitochondrial transcription factor A (TFAM). Loss of BMPR2-p53 signaling destabilizes mitochondrial DNA integrity and biogenesis causing adenosine triphosphate (ATP)-crises-mediated PAEC apoptosis which is associated with an inability to recover from hypoxia-induced PH (20). While, strictly speaking, p53 itself is a TF complex by auto-multimerization, fine-tuning of cellular effects depends on additional context-specific interaction partners (120). In the pulmonary vasculature, in response to oxidative stress and other DNA-damaging agents, p53 forms a transcriptionally active, vasculoprotective complex with PPARγ in PAEC, PASMC, and PAAF which is BMPR2-dependent (28). This is discussed further in the section on TF complexes of this review.

#### NF-kB

Strictly speaking, *nuclear factor kappa-light-chain-enhancer of activated B cells* (NF-κB) resembles a TF complex, best studied in cancer, that mediates transcription of proinflammatory cytokines and thus promotes unfavorable cell phenotypes (130). In advanced PAH, NF-κB is active in PAEC, PASMC and perivascular macrophages and lymphocytes of large and small pre-capillary vessels and is correlated with expression of pro-inflammatory cytokines (131). In PAEC, NF-κB contributes to leucocyte adhesion and inflammation facilitating EndMT (132). In contrast, genetic and pharmacological inhibition of NF-κB reversed and prevented experimental PAH in rodent models, respectively (133, 134). This indicates that targeting might be a reasonable therapeutic strategy.

#### TBX4

*T-box TF 4* (TBX4) is necessary in embryonal development and a gene mutation leads into an autosomal-dominant disorder called small patella syndrome (135). Kerstjens-Frederikse et al. showed that genetically depleted TBX4 is associated with childhood-onset PAH, which, with 0.7 cases per million, is an even rarer disease than PAH (136).

#### STAT3

Several physiological processes, like cell growth and apoptosis, are affected by the pro-survival TF *signal transducers and activators of transcription*-3 (STAT3) and an inhibition always leads to dramatic changes in biological processes. In PASMCs, Paulin et al. demonstrated that STAT3 activation induces proliferation and resistance to apoptosis by activating NFAT (137).

#### STAT1

Gairhe et al. showed that *Signal Transducer and Activator of Transcription 1* (STAT1) is elevated in PAECs with caveolin1 loss-of-function. This results in a proliferative, hypermigratory phenotype (138). Also Otsuki et al. showed, that PAECs stimulated with human endogenous retrovirus K (HERV-K) dUTPase have a TLR4-STAT1-dependent inflammatory response (132).

### TF superclass 7

*This superclass features an alpha-/beta-scaffold in the DNA-binding domain* (48).

#### SMAD3

SMADs, in particular phospho-SMAD1/5/8 are important downstream TF of BMPR2 signaling in the pulmonary vasculature (23). *SMAD family member 3* (SMAD3) is downregulated in lungs from PAH patients or animal models. Loss of SMAD3 is associated with a hyperproliferative and pro-migratory PASMC and PAEC phenotype in a myocardin-related transcription factor (MRTF)-dependent manner (139). In PAEC stimulated with HERV-K dUTPase, activation of SMAD3 can induce EndMT via SNAIL (132). More details about SMAD signaling can be found in the section on TF complexes of this review.

### TF superclass 8

*In this superclass, DNA binding occurs through β-sheets* (48). *A single TF from this superclass has been associated with PAH pathogenesis.*

#### HMGA1

*High Mobility Group AT-hook* 1 (HMGA1) is upregulated in PAECs of PAH patients, which is associated with a loss of BMPR2. By inducing SLUG expression, HMGA1 promotes EndMT of PAECs into an SMC-like mesenchymal phenotype in the vasculature of BMPR2-mutant PAH patients (54).

### Newly identified transcription factors with unknown impact

In addition to the above-listed TFs with well-established implications for PAH, there are other TFs that might contribute to the disease. A recent comprehensive analysis of chromatin remodeling in PAEC identified 18 novel TFs with differential activity in PAH compared with healthy control donors (more active in PAH: ATF1, ATF7, E4F1, CREB5, RFX3, RFX4, FOSL1, FOSL2, JUN, JUND, BATF; more active in controls: ARI3A, FOXG1, FOXJ3, FOXL1, TBX3, PITX2). These TFs are not discussed further in this review as the exact molecular mechanisms and associated pathophysiological ramifications remain to be elucidated (140).

### Pathogenic genetic transcription factor variants in pulmonary arterial hypertension

Specific variants in at least 22 genes have been associated with PAH pathogenesis (78, 105, 141, 142). Of these, six genes code for TFs, namely KLF2, SMAD1, SMAD4, SMAD9, SOX17, and TBX4. Variants in two of these genes show definitive associations with PAH pathogenesis as classified by independent expert panel working groups (Table 2).

## Transcription factor complexes: Basics

Finely tuned regulation of transcription requires sequence-specific DNA binding of TFs and co-factors. The combination of multiple TFs is termed combinational control. Cooperation between multiple copies of the same TF, or between different TFs, can stimulate transcriptional synergy in which the regulatory effect of TFs working together is greater than the sum of the individual TFs (143). Cooperative TFs typically generate transcriptional output through such multi-protein complexes (144). A distinction must be made between *(1)* complexes that consist of several TFs, i.e., individual TFs that have a potentially transcriptional modulatory effect on their own, and *(2)* complexes in which TFs are influenced in their function by cofactors. Depending on the binding partner and cell type, a single TF can thus influence various signaling pathways through complex formation (145). Whether a TF complex consisting of at least two TFs is referred to as a TF complex, TF dimer, or TF multimer, or to what extent a distinction is made between TF-TF complex and TF-co-factor complexes has not yet been defined uniformly.

## Transcription factor complexes in the pathogenesis of pulmonary arterial hypertension

**FIGURE 2:**
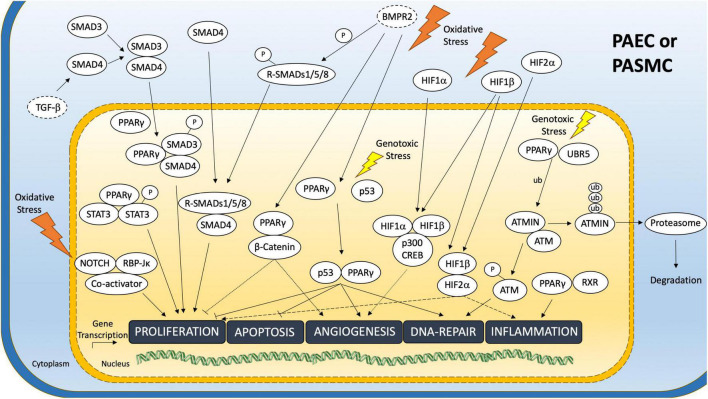
Transcription Factor Complexes in the Pulmonary Vasculature related to PAH. Overview of most relevant TF complex pathways in the pulmonary vasculature. TF complex formation upon complex inducing microenvironmental stimuli and downstream TF complex mediated gene transcription programs are shown. Yellow box represents nucleus. Blue box represents cell membrane. Dotted ovals indicate cell membrane anchored receptors. ub, Ubiquitin residue/Ubiquitination; –p, Phosphorylation.

Although a multitude of TF complexes are known to affect pulmonary cells, only a few of them have been identified to play a pivotal role in the pathogenesis of PAH or harbor therapeutic potential. A descriptive overview of the best-described TF complexes in PAEC and PASMC is given below and summarized in Figure 2.

### AP1 complex

*The activator protein 1* (AP1) TF complex, which is composed of c-JUN-c-FOS and c-JUN–c-JUN dimers, is regulated by many extracellular stimuli like peptide growth factors, pro-inflammatory cytokines, and other forms of cellular stress (146). In the vessel wall of lungs from IPAH patients, higher levels of total and phosphorylated c-FOS and c-JUN were detected, which results in an altered proliferative response in PASMCs mediated by the potent vasoconstrictor endothelin-1 (ET1) (147).

### NOTCH1/RBP-Jκ TF-complex

The transmembrane protein *Neurogenic locus notch homolog protein 1* (NOTCH1), which can be activated by extracellular ligands or hypoxia, releases the *Notch intracellular domain* (NICD) through proteolytic cleavage, which then translocates to the nucleus. There, NICD binds to the *Recombinant Signal Sequence Binding Protein J kappa* (RBP-Jκ) to form a heterodimeric TF complex. This complex positively influences the proliferation of PAEC and exerts anti-apoptotic effects (148). In addition, PAEC-PASMC contact, mediated by BMPR2-activated NOTCH1, induces transcription of endothelial regeneration genes, and coordinates the link between PAEC metabolism and chromatin remodeling to activate vascular homeostasis and repair in response to endothelial injury (149).

### Hypoxia-inducible factor complexes

*Hypoxia-inducible factor* (HIF) represents a TF complex that is highly responsive to subtle changes in the environmental oxygen content of the lung. The HIF complex is permanently formed and degraded under normoxic conditions (150) and HIF complex dynamics are finely tuned: With decreasing ambient oxygen content the complex is rapidly stabilized and is also degraded within minutes when reoxygenation occurs (150, 151). In the lung, HIF isoforms HIF1α and HIF2α, individually form a TF complex with HIF1β (also known as *aryl hydrocarbon receptor nuclear translocator*, ARNT) (152–154).

Under hypoxia, HIF1 (= HIF1α/HIF1β complex) recruits another co-factor, CREB/p300 to bind to the hypoxia-responsive element (HRE) in the promoter region of its target genes (151) to transcriptionally regulate angiogenesis, vascular tone and remodeling (150, 151, 155, 156).

HIF1 is the predominant hypoxia sensor in PASMC and promotes a hyperproliferative PASMC phenotype (157). HIF1α expression is increased in pulmonary arteries of PAH patients (158) and contributes to the hyperproliferation of PASMC by modulating the vascular tone through altered expression of membrane ion channels in PASMC (159, 160). HIF1 directly induces expression of angiogenetic genes like VEGF (156) via nitric oxide (NO) synthases 2 (NOS2) which synthesizes the most potent vasodilator NO (158, 161, 162). Under conditions of reduced NOS2 expression or impaired activity, the relaxing effect of NO on the PA vascular bed is attenuated thereby facilitating vascular remodeling and neointima formation via increased PASMC proliferation and resistance to apoptosis (163, 164). In addition to NO synthesis, Wang and Ying describe another mechanism influencing vascular tone: Loss of HIF1α induces expression of miRNA-543, which then downregulates Twist1 resulting in increased expression of the potent vasoconstrictor ET-1 in PASMC (44). Mitogenic effects of ET-1 have been shown to be associated with PA remodeling (165). Vascular remodeling in the lung is further facilitated by HIF1 via transcriptional repression of miRNA-223 in PASMCs leading to increased *PARP-1* expression (166), via a feedback loop with KLF5 (see above, 86) and by HIF1-dependent upregulation of plasminogen activator inhibitor-1 (PAI-1) (167) and Ras association domain-containing protein 1A (RASSF1A) to promote hypoxia-signaling to PASMC in PAH thereby likely conferring a cancer-like PASMC phenotype (168). Interestingly, PASMC proliferation caused by the transient HIF1 activation is attenuated by treatment with PPARγ activator rosiglitazone (169). HIF1α also mediates a metabolic shift to a cancer-like Warburg phenomenon in PAEC (158). Nevertheless, in PAEC HIF2α appears to be the predominant HIF isoform (147, 148).

HIF2α is increased in lung vascular ECs (LVECs) of IPAH patients which was associated with downregulation of HIF2α degrading enzyme prolyl hydroxylase domain protein 2 (PHD2). This resulted in induction of SNAI1/2 expression facilitating EndMT and formation of pulmonary vascular lesions (170). Endothelial-specific KO of PDH2 leads to experimental PH under normoxia which was dependent on HIF2α but not HIF1α (171). HIF2α also influences vascular resistance in the pulmonary vasculature. Mice with heterozygous global KO of HIF2α were protected from hypoxia-induced PH in an ET-1- and plasma catecholamine-dependent manner (172). Endothelial HIF2α disturbs EC NO homeostasis by upregulation of Arginase and mice with endothelial deletion of HIF2α were protected from hypoxia-induced PH (173). On the other hand, an activating mutation in the HIF2α gene is associated with erythrocytosis and pulmonary hypertension (174, 175), which, interestingly, seems to be mostly related to a phenotypic switch of PASMC but not PAEC (176).

It is likely that the HIF complex also interacts with additional TFs in the pulmonary vasculature to fine-tune hypoxia-associated gene expression. In this light, Palmer et al. suggest that HIF1α cooperates with activating Transcription Factor 1 (ATF-1) and/or CREB-1 either in the form of a complex or to functionally replace the two TFs in hypoxia (151). Additional interaction partners and their role in PA maintenance and remodeling remain to be elucidated.

### SMAD complexes

Dysfunction of the BMPR2 signal transduction is found in all forms of PAH (24, 177, 178). Normally, BMPR2 activation triggers a canonical signaling pathway resulting in phosphorylation of *Receptor*-regulated *Small Mothers Against Decapentaplegic Homolog Family* members (R-SMADs, SMAD 1/5/8) (23). Activated R-SMADs form a heteromeric complex with common mediators (Co-SMADs, SMAD4). The R-SMAD-Co-SMAD complex translocates into the nucleus (179). SMAD proteins are crucial for cell development, the transcription of specific vasculoprotective target genes (180) and growth regulation by activating the TGF-β superfamily. While R/Co-SMADs activate the TGF-β pathway, I-SMADs disrupt the TGF-β pathway. Disturbed SMAD signaling leads to increased MSX1, which seems to be associated with IPAH and HPAH pathogenesis (96). The BMPR2-Smad axis is a promising therapeutic target as SMAD signaling can be pharmacologically reactivated on the BMPR2 level by tacrolimus (24, 181).

### STAT3 homodimer

*Signal transducer and activators of transcription-3* (STAT3) is a cytoplasmatic transcription factor, which is activated in response to cytokines (IL-6), growth factors (PDGF), and agonists (ET1) and mediates its function as a homodimer (182). It plays an important role in regulating the expression of multiple proteins and TFs associated with the pathogenesis of PAH such as HIF1α, Pim1, and NFAT. STAT3 signaling confers a cancer-like, hyperproliferative, anti-apoptotic phenotype to PAH PASMC (183). Functionally, STAT3 promotes pro-inflammatory processes by increasing the recruitment of inflammatory cells through induction of interleukin-6 (IL-6). In addition, STAT3 activation increases proliferation and migration of vascular SMCs in response to vascular injuries (184). STAT3 also regulates the miR-cluster17/92 and miR-204, which regulates BMPR2 translation. Via induction of KLF5, STAT3 augments transcription of the anti-apoptotic gene BIRC5 (survivin) to increase PASMC proliferation (184, 185). Another way, STAT3 promotes a pro-proliferative PASMC phenotype found in PAH patients is by increasing PIM1 gene expression and Nuclear Factor of Activated T Cells 2 (NFATC2) activity (183, 185).

### YAP/TAZ/TEAD complex

Transcriptional co-regulators *Yes-associated protein 1* (YAP) and *Transcriptional Co-Activator with PDZ-Binding Motif* (TAZ, official gene symbol: WWTR1) form complexes with *TEA domain* (TEAD) transcription factors and function as mechanotransducers and -effectors of the Hippo signaling cascade (186). Altered mechanobiological properties are a well-established pathological feature of PAH and stiffening of the ECM initiates a vicious circle of vessel wall remodeling that is further promoting ECM rigidity [reviewed in: (187)]. In this context, Bertero et al. have shown that ECM remodeling activates YAP/TAZ, which then induces expression of miRNA-130/301 independent of TEAD (188). miRNA-130/301 then promoted PA collagen deposition, lysyl oxidase (LOX) activation with subsequent release of pro-fibrotic factors causing proliferation of PAEC, PASMC, and PAAF and subsequent vessel wall remodeling, ECM stiffening and thus further YAP/TAZ activation (188). In addition, pulmonary vascular stiffening-associated YAP/TAZ activation also promoted metabolic reprogramming of PAEC through direct transcriptional regulation of the key enzyme of glutaminolysis, GLS1 (189). YAP/TAZ activation also contributes to PAH severity by suppressing anti-inflammatory and vasodilatory cyclooxygenase-2 and prostaglandin F_1α_ in a TEAD-dependent fashion in PASMC (190).

### PPARγ/RXRα complex

*Peroxisome proliferator-activated receptors* (PPARs) belong to a family of nuclear hormone receptors called nuclear factors. Different PPAR isoforms (α, β/δ, γ) are ubiquitously expressed, while PPARγ represents the main isoform in pulmonary vascular cells (66, 191). PPARs usually bind to a nuclear receptor response element (NRRE) in the promoter region of their target genes in complex with a co-repressor or co-activator and a histone deacetylase. Interaction with PPAR ligands forces co-repressor dissociation to activate the transcription machinery (192). Likewise, co-activators heavily influence the cellular response of this TF-complex by chromatin acetylation thereby making it accessible to RNA polymerase II (61). Typically, PPARs form heterodimers with their canonical interaction partner, *retinoid X receptor* (RXR), to control transcription of target genes that play a critical role in energy balance, including triglyceride and fatty acid metabolism and glucose homeostasis: processes that are dysregulated in obesity, diabetes, and atherosclerosis (58, 60, 61, 193, 194). It is highly likely, that most anti-inflammatory and vasculoprotective PPARγ effects in the pulmonary vasculature for which no exclusive PPARγ interaction partners have been identified are mediated by the PPARγ/RXRα complex (34, 59, 194).

### PPARγ/MRN and PPARγ/UBR5 complexes

Although not a classical TF-TF complex, another DNA-associated PPARγ protein complex is of special interest for PAH pathogenesis. Upon genotoxic stress, PPARγ interacts with the DNA damage-sensing heterotrimer MRE11-RAD50-NBS1 (MRN) to facilitate DNA repair via the ATM pathway. This also requires the interaction of PPARγ with UBR5, an E3 ubiquitin-protein ligase, responsible for damage-associated degradation of ATM inhibitor (ATMIN) (70). Interestingly, the PPARγ-UBR5 interaction is disturbed in PAEC of PAH patients. This corresponds to an inability to activate the DNA damage response pathway upon genotoxic stress and to repair DNA damage (70).

### PPARγ/β-catenin complex

The protein β-catenin is normally involved in cell adhesion and gene transcription. In PAECs, PPARγ forms a BMPR2-mediated TF complex with β-catenin (PPARγ/β-catenin complex). In PAH patients with a dysfunctional BMPR2-signaling, the expression of PPARγ//β-catenin inducible vasculoprotective genes such as Apelin is reduced. Apelin-deficient PAECs are prone to apoptosis and promote PASMC proliferation (46).

### PPARγ/SMAD3 and PPARγ/STAT3 complexes

Two other interesting complexes are related to PASMC proliferation and metabolism: PPARγ/SMAD3 and PPARγ/STAT3. On the one hand, PPARγ inhibits the well-known canonical TGF-β1-pSMAD3/4 signaling pathway through interactions with SMAD3 and, on the other hand, the non-canonical TGF-β/STAT3-FoxO1 signaling, which is mostly unknown (33). Interestingly, the direct interaction between PPARγ-SMAD3 (cytoplasm) and PPARγ-STAT3 (nucleus) inhibits TGF-induced phosphorylation and shuttling of SMAD3/4 and STAT3/FoxO1 through pioglitazone which resulted in altered proliferation and metabolism (67).

### PPARγ/p53 complex

Under conditions of genotoxic stress PPARγ and p53 form a TF complex in various cell types (28, 195). In the pulmonary vasculature, PPARγ and p53 interact physically in all cell types across the vessel wall, namely PAEC, PASMC, and PA adventitial fibroblasts, to activate a vasculo-regenerative gene transcription program, which in PAEC is BMPR2-dependent (20, 28). Of note, the PPARγ-p53 TF complex can be harnessed pharmacologically as Nutlin-3, a p53-stabilizing compound, induces complex formation even under conditions of dysfunctional or lacking BMPR2, thereby salvaging impaired transcription of vasculoprotective genes including but not limited to genes promoting EC metabolism, survival, angiogenesis, and DNA repair (28). In a genetic PAH model with endothelial cell-specific BMPR2 knockout Nutlin-3 induces formation of the PPARγ-p53 complex and upregulation of complex target genes in lung microvascular EC was associated with reversal of persistent pulmonary hypertension, PA remodeling, and regeneration of pulmonary microvessels (28).

## Therapeutic potential of transcription factors in pulmonary arterial hypertension

Despite the many advances in recent decades, PAH remains a disease with a poor long-term prognosis. If left untreated, around 2–10% of patients die in the first year after diagnosis (196). Current therapies can only delay, but not prevent or reverse progression to right heart failure (11). Currently approved PAH-specific therapies target four different pathways: (1) the endothelin pathway promotes vasoconstriction and proliferation, therefore endothelin receptor blockers (ERA) are used, (2) prostacyclins or prostanoid receptor agonists directly promote vasodilatation and partially exert anti-proliferative effects, (3) activation of the NO-sGC-cGMP pathway has vasodilatory and anti-proliferative effects, and (4) in the subset of vasoresponsive PAH patients voltage-dependent calcium-channel blocker are used (196, 197). An early and upfront combination of these drugs is recommended to improve long-term outcomes (198). Thus, although long-term mortality has significantly improved, it remains high (199, 200).

Currently approved pharmacological options for PAH mainly influence the vascular tone. Therefore, current medication cannot significantly reverse the pathologically dysregulated signaling pathways that lead to vascular remodeling through inflammation, growth factor signaling, and metabolic dysfunction (10). New treatment options for PAH patients are therefore needed to further improve outcome.

In this light, TF-based therapies might pave way for reverse remodeling strategies. TFs are involved in numerous pathological conditions like cancer, diabetes, or cardiovascular diseases. However, TFs were long deemed “undruggable,” yet targeting transcription factors for therapy has become reality (201). Strategies include the use of small molecule compounds to modulate TF activity, e.g., by inhibition of TF (-co-factor) complex formation or DNA binding or promotion of TF degradation [reviewed in (202)]. For some diseases, TF targeting therapies are clinically well established like TZD therapy in type 2 diabetes mellitus (203). For PAH, multiple novel compounds are currently in clinical trials with promising candidate TF pathways still in preclinical phases (204).

One such candidate is FOXO1 (205). Loss of FOXO1 function in PASMCs promoted a disease phenotype *in vitro* and *in vivo* and caused experimental PAH. On the other hand, pharmacological activation of FOXO1 was associated with reconstitution of a healthy PASMC phenotype and reversal of experimental PH (93). The multitude of routes and options for pharmacological FOXO1 activation (206) augurs well for FOXO1-based PAH treatment strategies (93, 206).

HIF has been a TF of interest as a therapeutic target for PAH for many years (150, 157). Early evidence indicated that pharmacological inhibition of HIF1 and HIF2α attenuated hypoxia-induced pulmonary hypertension, RV hypertrophy and PA remodeling by inhibiting intracellular Ca^2+^ release and pH changes upon hypoxia in PASMC (207). In general, at least 12 different pharmacological inhibitors of HIF1 and HIF2α were able to attenuate, prevent or reverse experimental pulmonary hypertension in rats or mice [reviewed in (157)] and multiple strategies appear feasible: Besides pharmacological inhibition of HIF signaling, destabilization of HIF via activation of HIF-degrading enzyme cascades or disruption of HIF complexes have all shown promising results as potential therapeutic strategies in experimental PH (and, partly, in ECs isolated from PAH patients) (208–210). In addition, HIF augurs well for novel combination therapies since pharmacological inhibition of HIF2α with simultaneous activation of p53 was more effective in reversing experimental PH and vascular remodeling than either treatment alone (211). This is particularly interesting, as pharmacological activation of p53 has been shown to reverse experimental PH by PASMC- (129) and PAEC (28)-specific mechanisms (see below).

The YAP/TAZ/TEAD pathway can also be harnessed as a therapeutic target in PAH. Pharmacological blunting of YAP/TAZ activation by glutaminase inhibitors, LOX inhibitors, ApoE activators or gene therapy using adeno-associated viruses expressing shRNA against the newly identified YAP/TAZ upstream regulator HSP110 attenuated or reversed experimental PH in rodent models (188, 189, 212).

Despite recent evidence that emphasizes the beneficial role of HIF2α inhibition (in endothelial cells) as a therapeutic target for pulmonary hypertension (208–210), thorough selection of patients to test proof-of-concept of these results in humans will be necessary as endothelial HIF2α appears to be crucial for vascular survival and maintenance of a functional alveolar structure (213, 214). As an alternative, targeting HIF1 in PASMC might be a reasonable approach (157).

As mentioned before, PPARγ is pivotal for maintaining pulmonary vascular homeostasis via complex formation with various interaction partners. PPARγ activation through endogenous ligands or pharmacological compounds has been shown to convey a broad spectrum of beneficial functions in the pulmonary vasculature from facilitation of normal cell signaling to maintaining pulmonary vascular cell homeostasis and promoting reverse remodeling of pathological vascular changes associated with PAH (28, 32–34, 46, 53, 63, 66, 67, 69, 215–219). In PAH studies, pharmacological activation of PPARγ is achieved by using thiazolidinediones (TZD, including Rosiglitazone and Pioglitazone), a class of drugs that has been under scrutiny for some time due to unwanted and potentially harmful side effects (34).

Earlier studies mostly used Rosiglitazone showing beneficial effects in various animal models of PH (53, 66, 216, 219). The first evidence in PAH came from Hansmann et al. who showed a complete reversal of right ventricular and pulmonary arterial remodeling by inhibition of proliferation and promotion of insulin sensitization in PASMC (66, 216). This was further substantiated in additional PH animal models by Liu et al. (219) as well as Bertero et al. (53). In a PAEC-dependent mechanism, Rosiglitazone restored miR-98 expression to attenuate ET-1-mediated hypoxia-induced PH (220). Due to the more favorable side effect profile, recent PH studies have used Pioglitazone (34). Pioglitazone also reversed PA and RV remodeling through beneficial effects on PASMC and cardiomyocytes (32, 33, 67, 217) by inhibiting canonical and non-canonical TGFβ1 signaling (33, 67), restoring mitochondrial homeostasis and improving cellular energy production by optimization of β-oxidation and glucose utilization (32).

Recently, we have also shown that PPARγ signaling is essential for Nutlin-3-mediated vasculoregeneration by modulating PAEC-protective p53 signaling (28). The small molecule compound Nutlin, currently in clinical trials for various cancers (221–223), induces activation of the PPARγ/p53 complex and a vasculo-regenerative gene transcription program in PAEC, PASMC, and PAAF (28). This resulted in reversal of persistent pulmonary hypertension in mice with endothelial-specific loss of BMPR2 via restoration of endothelial function and regeneration of pulmonary microvessels (28). In PAECs harboring BMPR2 mutations that were isolated from patients with PAH, Nutlin-induced PPARγ/p53 target genes facilitated the repair of prevalent DNA damage (28). In PASMC-based PH models, Nutlin-3 was also successful in preventing and reversing experimental PH by inhibiting PASMC proliferation through induction of a quasi-senescent phenotype (129). Since the PPARγ/p53 TF complex is also formed in PASMC (28) it would be interesting to see to which extent the beneficial effects of Nutlin-3 on PASMC are mediated by PPARγ/p53 complex target genes.

In addition to the direct targeting of TFs, the therapeutic potential of modulating TF co-factors or epigenetic factors which alter TF DNA is currently being investigated [reviewed in (45)].

In light of the recent advances in molecular strategies to modulate TF function, it appears to be only a matter of time before TF-based therapies will become a clinical reality in PAH treatment regimens (35).

## Conclusion

A growing body of evidence highlights the central role of TFs in the pathogenesis of PAH. Currently approved therapies mainly modulate vascular tone, but fail to significantly reverse pathological vascular changes in pulmonary arteries and microvessels or the right ventricle/heart. Dysregulation of TF function is closely associated with pathological remodeling of the pulmonary vasculature and the right heart. Multiple TFs have been identified that are related to either maintaining pulmonary vessel homeostasis or, if dysfunctional, contributing to vascular pathology. Even well-studied, pathways commonly dysbalanced in PAH such as BMPR2 and TGFβ signaling, often disembogue in a highly intertwined network of downstream TFs. These TFs often form complex protein-protein interaction networks (e.g., PPARγ) to elicit cell-specific, in part opposing functions, adding complexity.

However, critical knowledge gaps remain. Most importantly, available data suggest that TFs operate through elaborate networks comprising multiple TFs and Co-factors (28, 53, 70). Investigation of individual TFs may not fully reflect their biological function in the pulmonary vasculature and how they integrate a multitude of intracellular and extracellular signals. Therefore, additional systems biology approaches are needed to dissect the pathobiology of complex TF networks. An additional layer of complexity is added by chromatin remodeling phenomena in PAH, which directly affect TF activity (84, 140). In the pulmonary vasculature, it is thus necessary (a) to fully understand the underlying epigenetic mechanisms that facilitate three-dimensional chromatin conformation and accessibility and (b) how exactly epigenetic modifications affect TF networks. This is especially important in light of epigenetic modifiers emerging as druggable targets in PAH (224, 225).

While current data suggest that transcriptional dysfunction is an early event in PAH pathogenesis (4, 226, 227), the spatial resolution of TF dysfunction is less understood. Even though there is growing evidence that (microvascular) endothelial dysfunction precedes the pathological changes in PASMC and PAAF (228), it remains unclear what microniche-specific factors contribute to cell-type specific TF functions. In this regard, the application of single-cell epigenomics and multi-omics technologies will help uncover cell-type specific TF networks.

Therefore, the characterization of molecular TF functions, binding partners, and modes of action are essential for understanding PAH pathogenesis and identification of new therapeutic targets. Current experimental TF-based therapeutic strategies focus on modulating individual TF function, stability, or TF interaction partner network formation with very promising results.

Although specific targeting of dysregulated TF pathways in PAH is advantageous over the currently available broad and rather symptomatic therapeutic approaches, off-target effects need to be mitigated when using systemic drug strategies (229). Hence, utilizing gene therapy approaches with high selectivity (tropism) for specific pulmonary vascular cell types might be useful to overcome this (230, 231).

In summary, recent advances in our understanding of the underlying molecular mechanisms as well as tailored modulation of TF function pave the way for TF-based vasculo-regenerative or reverse remodeling therapies. The clinical usability of TF-based therapies needs to be validated in upcoming clinical trials.

## Author contributions

CM, JR, and JKl reviewed the literature and collected the data. CM made the illustrations and drafted the manuscript with support of JKl, JKö, and JKH. LH and HK helped to revise the manuscript and contributed to important intellectual content. JKö and JKH were responsible for oversight, design, manuscript preparation, and revision. All authors have read and approved the final manuscript.

## References

[1] Hoeper MM Kramer T Pan Z Eichstaedt CA Spiesshoefer J Benjamin N Mortality in pulmonary arterial hypertension: prediction by the 2015 European pulmonary hypertension guidelines risk stratification model. *Eur Respir J.* (2017) 50:1700740. 10.1183/13993003.00740-2017 28775047

[2] Badesch DB Champion HC Gomez Sanchez MA Hoeper MM Loyd JE Manes A Diagnosis and assessment of pulmonary arterial hypertension. *J Am Coll Cardiol.* (2009) 54:S55–66. 10.1016/j.jacc.2009.04.011 19555859

[3] Simonneau G Montani D Celermajer DS Denton CP Gatzoulis MA Krowka M Haemodynamic definitions and updated clinical classification of pulmonary hypertension. *Eur Respir J.* (2019) 53:1801913. 10.1183/13993003.01913-2018 30545968 PMC6351336

[4] Humbert M Guignabert C Bonnet S Dorfmuller P Klinger JR Nicolls MR Pathology and pathobiology of pulmonary hypertension: state of the art and research perspectives. *Eur Respir J.* (2019) 53:1801887. 10.1183/13993003.01887-2018 30545970 PMC6351340

[5] Ranchoux B Antigny F Rucker-Martin C Hautefort A Pechoux C Bogaard HJ Endothelial-to-mesenchymal transition in pulmonary hypertension. *Circulation.* (2015) 131:1006–18. 10.1161/CIRCULATIONAHA.114.008750 25593290

[6] Ricard N Tu L Le Hiress M Huertas A Phan C Thuillet R Increased pericyte coverage mediated by endothelial-derived fibroblast growth factor-2 and interleukin-6 is a source of smooth muscle-like cells in pulmonary hypertension. *Circulation.* (2014) 129:1586–97. 10.1161/CIRCULATIONAHA.113.007469 24481949

[7] Crnkovic S Marsh LM El Agha E Voswinckel R Ghanim B Klepetko W Resident cell lineages are preserved in pulmonary vascular remodeling. *J Pathol.* (2018) 244:485–98. 10.1002/path.5044 29359814 PMC5903372

[8] Tian W Jiang X Sung YK Shuffle E Wu TH Kao PN Phenotypically silent bone morphogenetic protein receptor 2 mutations predispose rats to inflammation-induced pulmonary arterial hypertension by enhancing the risk for neointimal transformation. *Circulation.* (2019) 140:1409–25. 10.1161/CIRCULATIONAHA.119.040629 31462075 PMC6803052

[9] LeVarge BL. Prostanoid therapies in the management of pulmonary arterial hypertension. *Ther Clin Risk Manag.* (2015) 11:535–47. 10.2147/TCRM.S75122 25848300 PMC4386780

[10] Galie N Ghofrani AH. New horizons in pulmonary arterial hypertension therapies. *Eur Respir Rev.* (2013) 22:503–14. 10.1183/09059180.00006613 24293466 PMC9639192

[11] Spiekerkoetter E Kawut SM de Jesus Perez VA. New and emerging therapies for pulmonary arterial hypertension. *Annu Rev Med.* (2019) 70:45–59. 10.1146/annurev-med-041717-085955 30216732 PMC7735523

[12] Dannewitz Prosseda S Ali MK Spiekerkoetter E. Novel advances in modifying BMPR2 signaling in PAH. *Genes.* (2020) 12:8. 10.3390/genes12010008 33374819 PMC7824173

[13] Hennigs JK Luneburg N Stage A Schmitz M Korbelin J Harbaum L The P2-receptor-mediated Ca(2+) signalosome of the human pulmonary endothelium - implications for pulmonary arterial hypertension. *Purinergic Signal.* (2019) 15:299–311. 10.1007/s11302-019-09674-1 31396838 PMC6737170

[14] Pousada G Lupo V Castro-Sanchez S Alvarez-Satta M Sanchez-Monteagudo A Baloira A Molecular and functional characterization of the BMPR2 gene in pulmonary arterial hypertension. *Sci Rep.* (2017) 7:1923. 10.1038/s41598-017-02074-8 28507310 PMC5432510

[15] Rabinovitch M. Molecular pathogenesis of pulmonary arterial hypertension. *J Clin Invest.* (2012) 122:4306–13. 10.1172/JCI60658 23202738 PMC3533531

[16] Atkinson C Stewart S Upton PD Machado R Thomson JR Trembath RC Primary pulmonary hypertension is associated with reduced pulmonary vascular expression of type II bone morphogenetic protein receptor. *Circulation.* (2002) 105:1672–8. 10.1161/01.cir.0000012754.72951.3d 11940546

[17] Evans JD Girerd B Montani D Wang XJ Galie N Austin ED BMPR2 mutations and survival in pulmonary arterial hypertension: an individual participant data meta-analysis. *Lancet Respir Med.* (2016) 4:129–37. 10.1016/S2213-2600(15)00544-5 26795434 PMC4737700

[18] Lavoie JR Ormiston ML Perez-Iratxeta C Courtman DW Jiang B Ferrer E Proteomic analysis implicates translationally controlled tumor protein as a novel mediator of occlusive vascular remodeling in pulmonary arterial hypertension. *Circulation.* (2014) 129:2125–35. 10.1161/CIRCULATIONAHA.114.008777 24657995

[19] Richter A Yeager ME Zaiman A Cool CD Voelkel NF Tuder RM. Impaired transforming growth factor-beta signaling in idiopathic pulmonary arterial hypertension. *Am J Respir Crit Care Med.* (2004) 170:1340–8. 10.1164/rccm.200311-1602OC 15361368

[20] Diebold I Hennigs JK Miyagawa K Li CG Nickel NP Kaschwich M BMPR2 preserves mitochondrial function and DNA during reoxygenation to promote endothelial cell survival and reverse pulmonary hypertension. *Cell Metab.* (2015) 21:596–608. 10.1016/j.cmet.2015.03.010 25863249 PMC4394191

[21] Feng YX Liu D Sun ML Jiang X Sun N Mao YM BMPR2 germline mutation in chronic thromboembolic pulmonary hypertension. *Lung.* (2014) 192:625–7. 10.1007/s00408-014-9580-y 24728306

[22] Chen NY D Collum S Luo F Weng T Le TT M Hernandez A Macrophage bone morphogenic protein receptor 2 depletion in idiopathic pulmonary fibrosis and Group III pulmonary hypertension. *Am J Physiol Lung Cell Mol Physiol.* (2016) 311:L238–54. 10.1152/ajplung.00142.2016 27317687 PMC6425517

[23] Andruska A Spiekerkoetter E. Consequences of BMPR2 deficiency in the pulmonary vasculature and beyond: contributions to pulmonary arterial hypertension. *Int J Mol Sci.* (2018) 19:2499. 10.3390/ijms19092499 30149506 PMC6165502

[24] Spiekerkoetter E Tian X Cai J Hopper RK Sudheendra D Li CG FK506 activates BMPR2, rescues endothelial dysfunction, and reverses pulmonary hypertension. *J Clin Invest.* (2013) 123:3600–13. 10.1172/JCI65592 23867624 PMC3726153

[25] Yung LM Yang P Joshi S Augur ZM Kim SSJ Bocobo GA ACTRIIA-Fc rebalances activin/GDF versus BMP signaling in pulmonary hypertension. *Sci Transl Med.* (2020) 12:eaaz5660. 10.1126/scitranslmed.aaz5660 32404506 PMC8259900

[26] Long L Ormiston ML Yang X Southwood M Graf S Machado RD Selective enhancement of endothelial BMPR-II with BMP9 reverses pulmonary arterial hypertension. *Nat Med.* (2015) 21:777–85. 10.1038/nm.3877 26076038 PMC4496295

[27] Nickel NP Spiekerkoetter E Gu M Li CG Li H Kaschwich M Elafin reverses pulmonary hypertension via caveolin-1-dependent bone morphogenetic protein signaling. *Am J Respir Crit Care Med.* (2015) 191:1273–86. 10.1164/rccm.201412-2291OC 25853696 PMC4476518

[28] Hennigs JK Cao A Li CG Shi M Mienert J Miyagawa K PPARgamma-p53-mediated vasculoregenerative program to reverse pulmonary hypertension. *Circ Res.* (2021) 128:401–18. 10.1161/CIRCRESAHA.119.316339 33322916 PMC7908816

[29] Humbert M McLaughlin V Gibbs JSR Gomberg-Maitland M Hoeper MM Preston IR Sotatercept for the treatment of pulmonary arterial hypertension. *N Engl J Med.* (2021) 384:1204–15. 10.1056/NEJMoa2024277 33789009

[30] Spiekerkoetter E Sung YK Sudheendra D Bill M Aldred MA van de Veerdonk MC Low-Dose FK506 (tacrolimus) in end-stage pulmonary arterial hypertension. *Am J Respir Crit Care Med.* (2015) 192:254–7. 10.1164/rccm.201411-2061LE 26177174 PMC4532822

[31] Spiekerkoetter E Sung YK Sudheendra D Scott V Del Rosario P Bill M Randomised placebo-controlled safety and tolerability trial of FK506 (tacrolimus) for pulmonary arterial hypertension. *Eur Respir J.* (2017) 50:1602449. 10.1183/13993003.02449-2016 28893866

[32] Legchenko E Chouvarine P Borchert P Fernandez-Gonzalez A Snay E Meier M PPARgamma agonist pioglitazone reverses pulmonary hypertension and prevents right heart failure via fatty acid oxidation. *Sci Transl Med.* (2018) 10:eaao0303. 10.1126/scitranslmed.aao0303 29695452

[33] Calvier L Chouvarine P Legchenko E Kokeny G Mozes MM Hansmann G. Chronic TGF-beta1 signaling in pulmonary arterial hypertension induces sustained canonical smad3 pathways in vascular smooth muscle cells. *Am J Respir Cell Mol Biol.* (2019) 61:121–3. 10.1165/rcmb.2018-0275LE 31259625

[34] Hansmann G Calvier L Risbano MG Chan SY. Activation of the metabolic master regulator PPARgamma: a potential pioneering therapy for pulmonary arterial hypertension. *Am J Respir Cell Mol Biol.* (2020) 62:143–56. 10.1165/rcmb.2019-0226PS 31577451 PMC6993553

[35] Olschewski A Berghausen EM Eichstaedt CA Fleischmann BK Grunig E Grunig G Pathobiology, pathology and genetics of pulmonary hypertension: update from the Cologne consensus conference 2018. *Int J Cardiol.* (2018) 272S:4–10. 10.1016/j.ijcard.2018.09.070 30314839

[36] Vaquerizas JM Kummerfeld SK Teichmann SA Luscombe NM. A census of human transcription factors: function, expression and evolution. *Nat Rev Genet.* (2009) 10:252–63. 10.1038/nrg2538 19274049

[37] Xie Z Hu S Qian J Blackshaw S Zhu H. Systematic characterization of protein-DNA interactions. *Cell Mol Life Sci.* (2011) 68:1657–68. 10.1007/s00018-010-0617-y 21207099 PMC11115113

[38] Fulton DL Sundararajan S Badis G Hughes TR Wasserman WW Roach JC TFCat: the curated catalog of mouse and human transcription factors. *Genome Biol.* (2009) 10:R29. 10.1186/gb-2009-10-3-r29 19284633 PMC2691000

[39] Spitz F Furlong EE. Transcription factors: from enhancer binding to developmental control. *Nat Rev Genet.* (2012) 13:613–26. 10.1038/nrg3207 22868264

[40] Geertz M Shore D Maerkl SJ. Massively parallel measurements of molecular interaction kinetics on a microfluidic platform. *Proc Natl Acad Sci U.S.A.* (2012) 109:16540–5. 10.1073/pnas.1206011109 23012409 PMC3478601

[41] Lambert SA Jolma A Campitelli LF Das PK Yin Y Albu M The human transcription factors. *Cell.* (2018) 172:650–65. 10.1016/j.cell.2018.01.029 29425488 PMC12908702

[42] Han J Kaufman RJ. Physiological/pathological ramifications of transcription factors in the unfolded protein response. *Genes Dev.* (2017) 31:1417–38. 10.1101/gad.297374.117 28860159 PMC5588925

[43] Martin-Martin N Carracedo A Torrano V. Metabolism and transcription in cancer: merging two classic tales. *Front Cell Dev Biol.* (2017) 5:119. 10.3389/fcell.2017.00119 29354634 PMC5760552

[44] Wang CC Ying L Barnes EA Adams ES Kim FY Engel KW Pulmonary artery smooth muscle cell HIF-1alpha regulates endothelin expression via microRNA-543. *Am J Physiol Lung Cell Mol Physiol.* (2018) 315:L422–31. 10.1152/ajplung.00475.2017 29745253 PMC6172620

[45] Pullamsetti SS Perros F Chelladurai P Yuan J Stenmark K. Transcription factors, transcriptional coregulators, and epigenetic modulation in the control of pulmonary vascular cell phenotype: therapeutic implications for pulmonary hypertension (2015 Grover conference series). *Pulm Circ.* (2016) 6:448–64. 10.1086/688908 28090287 PMC5210074

[46] Alastalo TP Li M Perez Vde J Pham D Sawada H Wang JK Disruption of PPARgamma/beta-catenin-mediated regulation of apelin impairs BMP-induced mouse and human pulmonary arterial EC survival. *J Clin Invest.* (2011) 121:3735–46. 10.1172/JCI43382 21821917 PMC3163943

[47] Yeligar SM Kang BY Bijli KM Kleinhenz JM Murphy TC Torres G PPARgamma regulates mitochondrial structure and function and human pulmonary artery smooth muscle cell proliferation. *Am J Respir Cell Mol Biol.* (2018) 58:648–57. 10.1165/rcmb.2016-0293OC 29182484 PMC5946324

[48] Wingender E Schoeps T Haubrock M Krull M Donitz J. TFClass: expanding the classification of human transcription factors to their mammalian orthologs. *Nucleic Acids Res.* (2018) 46:D343–7. 10.1093/nar/gkx987 29087517 PMC5753292

[49] Klemm DJ Majka SM Crossno JT Jr Psilas JC Reusch JE Garat CV. Reduction of reactive oxygen species prevents hypoxia-induced CREB depletion in pulmonary artery smooth muscle cells. *J Cardiovasc Pharmacol.* (2011) 58:181–91. 10.1097/FJC.0b013e31821f2773 21562428 PMC3155008

[50] Karlsson C Jonsson M Asp J Brantsing C Kageyama R Lindahl A. Notch and HES5 are regulated during human cartilage differentiation. *Cell Tissue Res.* (2007) 327:539–51. 10.1007/s00441-006-0307-0 17093926

[51] Li X Zhang X Leathers R Makino A Huang C Parsa P Notch3 signaling promotes the development of pulmonary arterial hypertension. *Nat Med.* (2009) 15:1289–97. 10.1038/nm.2021 19855400 PMC2780347

[52] Kim J Kang Y Kojima Y Lighthouse JK Hu X Aldred MA An endothelial apelin-FGF link mediated by miR-424 and miR-503 is disrupted in pulmonary arterial hypertension. *Nat Med.* (2013) 19:74–82. 10.1038/nm.3040 23263626 PMC3540168

[53] Bertero T Lu Y Annis S Hale A Bhat B Saggar R Systems-level regulation of microRNA networks by miR-130/301 promotes pulmonary hypertension. *J Clin Invest.* (2014) 124:3514–28. 10.1172/JCI74773 24960162 PMC4109523

[54] Hong Z Chen KH DasGupta A Potus F Dunham-Snary K Bonnet S MicroRNA-138 and MicroRNA-25 down-regulate mitochondrial calcium uniporter, causing the pulmonary arterial hypertension cancer phenotype. *Am J Respir Crit Care Med.* (2017) 195:515–29. 10.1164/rccm.201604-0814OC 27648837 PMC5378421

[55] Zhang C Ma C Zhang L Zhang L Zhang F Ma M MiR-449a-5p mediates mitochondrial dysfunction and phenotypic transition by targeting Myc in pulmonary arterial smooth muscle cells. *J Mol Med.* (2019) 97:409–22. 10.1007/s00109-019-01751-7 30715622

[56] Mammoto T Muyleart M Konduri GG Mammoto A. Twist1 in hypoxia-induced pulmonary hypertension through transforming growth factor-beta-Smad signaling. *Am J Respir Cell Mol Biol.* (2018) 58:194–207. 10.1165/rcmb.2016-0323OC 28915063

[57] Hopper RK Moonen JR Diebold I Cao A Rhodes CJ Tojais NF In pulmonary arterial hypertension, reduced BMPR2 promotes endothelial-to-mesenchymal transition via HMGA1 and its target slug. *Circulation.* (2016) 133:1783–94. 10.1161/CIRCULATIONAHA.115.020617 27045138 PMC4856565

[58] Green DE Sutliff RL Hart CM. Is peroxisome proliferator-activated receptor gamma (PPARgamma) a therapeutic target for the treatment of pulmonary hypertension? *Pulm Circ.* (2011) 1:33–47. 10.4103/2045-8932.78101 21547012 PMC3085428

[59] Hansmann G Zamanian RT. PPARgamma activation: a potential treatment for pulmonary hypertension. *Sci Transl Med.* (2009) 1:12s14. 10.1126/scitranslmed.3000267 20371457

[60] Bradley EA Bradley D. Pulmonary arterial hypertension and insulin resistance. *J Mol Genet Med.* (2014) 2:15. 10.4172/1747-0862.S1-015 29552090 PMC5856452

[61] Yu S Reddy JK. Transcription coactivators for peroxisome proliferator-activated receptors. *Biochim Biophys Acta.* (2007) 1771:936–51. 10.1016/j.bbalip.2007.01.008 17306620

[62] Ameshima S Golpon H Cool CD Chan D Vandivier RW Gardai SJ Peroxisome proliferator-activated receptor gamma (PPARgamma) expression is decreased in pulmonary hypertension and affects endothelial cell growth. *Circ Res.* (2003) 92:1162–9. 10.1161/01.RES.0000073585.50092.14 12714563

[63] Guignabert C Alvira CM Alastalo TP Sawada H Hansmann G Zhao M Tie2-mediated loss of peroxisome proliferator-activated receptor-gamma in mice causes PDGF receptor-beta-dependent pulmonary arterial muscularization. *Am J Physiol Lung Cell Mol Physiol.* (2009) 297:L1082–90. 10.1152/ajplung.00199.2009 19801450 PMC2793182

[64] Bijli KM Kleinhenz JM Murphy TC Kang BY Adesina SE Sutliff RL Peroxisome proliferator-activated receptor gamma depletion stimulates Nox4 expression and human pulmonary artery smooth muscle cell proliferation. *Free Radic Biol Med.* (2015) 80:111–20. 10.1016/j.freeradbiomed.2014.12.019 25557278 PMC4355175

[65] Green DE Murphy TC Kang BY Searles CD Hart CM. PPARgamma ligands attenuate hypoxia-induced proliferation in human pulmonary artery smooth muscle cells through modulation of MicroRNA-21. *PLoS One.* (2015) 10:e0133391. 10.1371/journal.pone.0133391 26208095 PMC4514882

[66] Hansmann G de Jesus Perez VA Alastalo TP Alvira CM Guignabert C Bekker JM An antiproliferative BMP-2/PPARgamma/apoE axis in human and murine SMCs and its role in pulmonary hypertension. *J Clin Invest.* (2008) 118:1846–57. 10.1172/JCI32503 18382765 PMC2276393

[67] Calvier L Chouvarine P Legchenko E Hoffmann N Geldner J Borchert P PPARgamma links BMP2 and TGFbeta1 pathways in vascular smooth muscle cells, regulating cell proliferation and glucose metabolism. *Cell Metab.* (2017) 25:1118–34.e7. 10.1016/j.cmet.2017.03.011 28467929

[68] Chakraborty R Saddouk FZ Carrao AC Krause DS Greif DM Martin KA. Promoters to study vascular smooth muscle. *Arterioscler Thromb Vasc Biol.* (2019) 39:603–12. 10.1161/ATVBAHA.119.312449 30727757 PMC6527360

[69] Vattulainen-Collanus S Akinrinade O Li M Koskenvuo M Li CG Rao SP Loss of PPARgamma in endothelial cells leads to impaired angiogenesis. *J Cell Sci.* (2016) 129:693–705. 10.1242/jcs.169011 26743080 PMC5108588

[70] Li CG Mahon C Sweeney NM Verschueren E Kantamani V Li D PPARgamma interaction with UBR5/ATMIN promotes DNA repair to maintain endothelial homeostasis. *Cell Rep.* (2019) 26:1333–43.e7. 10.1016/j.celrep.2019.01.013 30699358 PMC6436616

[71] Tian J Smith A Nechtman J Podolsky R Aggarwal S Snead C Effect of PPARgamma inhibition on pulmonary endothelial cell gene expression: gene profiling in pulmonary hypertension. *Physiol Genomics.* (2009) 40:48–60. 10.1152/physiolgenomics.00094.2009 19825830 PMC2807211

[72] Kokudo T Suzuki Y Yoshimatsu Y Yamazaki T Watabe T Miyazono K. Snail is required for TGFbeta-induced endothelial-mesenchymal transition of embryonic stem cell-derived endothelial cells. *J Cell Sci.* (2008) 121:3317–24. 10.1242/jcs.028282 18796538

[73] Bhattacharyya S Fang F Tourtellotte W Varga J. Egr-1: new conductor for the tissue repair orchestra directs harmony (regeneration) or cacophony (fibrosis). *J Pathol.* (2013) 229:286–97. 10.1002/path.4131 23132749 PMC3965177

[74] Kwapiszewska G Chwalek K Marsh LM Wygrecka M Wilhelm J Best J BDNF/TrkB signaling augments smooth muscle cell proliferation in pulmonary hypertension. *Am J Pathol.* (2012) 181:2018–29. 10.1016/j.ajpath.2012.08.028 23058367

[75] Dickinson MG Kowalski PS Bartelds B Borgdorff MA van der Feen D Sietsma H A critical role for Egr-1 during vascular remodelling in pulmonary arterial hypertension. *Cardiovasc Res.* (2014) 103:573–84. 10.1093/cvr/cvu169 25028387

[76] Yu X Chen X Zheng XD Zhang J Zhao X Liu Y Growth differentiation factor 11 promotes abnormal proliferation and angiogenesis of pulmonary artery endothelial cells. *Hypertension.* (2018) 71:729–41. 10.1161/HYPERTENSIONAHA.117.10350 29463625

[77] Doddaballapur A Michalik KM Manavski Y Lucas T Houtkooper RH You X Laminar shear stress inhibits endothelial cell metabolism via KLF2-mediated repression of PFKFB3. *Arterioscler Thromb Vasc Biol.* (2015) 35:137–45. 10.1161/ATVBAHA.114.304277 25359860

[78] Eichstaedt CA Song J Viales RR Pan Z Benjamin N Fischer C First identification of *Kruppel*-like factor 2 mutation in heritable pulmonary arterial hypertension. *Clin Sci.* (2017) 131:689–98. 10.1042/CS20160930 28188237

[79] Eichstaedt CA Sassmannshausen Z Shaukat M Cao D Xanthouli P Gall H Gene panel diagnostics reveals new pathogenic variants in pulmonary arterial hypertension. *Respir Res.* (2022) 23:74. 10.1186/s12931-022-01987-x 35346192 PMC8962083

[80] Sindi HA Russomanno G Satta S Abdul-Salam VB Jo KB Qazi-Chaudhry B Therapeutic potential of KLF2-induced exosomal microRNAs in pulmonary hypertension. *Nat Commun.* (2020) 11:1185. 10.1038/s41467-020-14966-x 32132543 PMC7055281

[81] Rhodes CJ Wharton J Boon RA Roexe T Tsang H Wojciak-Stothard B Reduced microRNA-150 is associated with poor survival in pulmonary arterial hypertension. *Am J Respir Crit Care Med.* (2013) 187:294–302. 10.1164/rccm.201205-0839OC 23220912

[82] Chandra SM Razavi H Kim J Agrawal R Kundu RK de Jesus Perez V Disruption of the apelin-APJ system worsens hypoxia-induced pulmonary hypertension. *Arterioscler Thromb Vasc Biol.* (2011) 31:814–20. 10.1161/ATVBAHA.110.219980 21233449 PMC3113525

[83] Ban Y Liu Y Li Y Zhang Y Xiao L Gu Y S-nitrosation impairs KLF4 activity and instigates endothelial dysfunction in pulmonary arterial hypertension. *Redox Biol.* (2019) 21:101099. 10.1016/j.redox.2019.101099 30660098 PMC6348764

[84] Moonen JR Chappell J Shi M Shinohara T Li D Mumbach MR KLF4 recruits SWI/SNF to increase chromatin accessibility and reprogram the endothelial enhancer landscape under laminar shear stress. *Nat Commun.* (2022) 13:4941. 10.1038/s41467-022-32566-9 35999210 PMC9399231

[85] Courboulin A Tremblay VL Barrier M Meloche J Jacob MH Chapolard M *Kruppel*-like factor 5 contributes to pulmonary artery smooth muscle proliferation and resistance to apoptosis in human pulmonary arterial hypertension. *Respir Res.* (2011) 12:128. 10.1186/1465-9921-12-128 21951574 PMC3193170

[86] Li X He Y Xu Y Huang X Liu J Xie M KLF5 mediates vascular remodeling via HIF-1alpha in hypoxic pulmonary hypertension. *Am J Physiol Lung Cell Mol Physiol.* (2016) 310:L299–310. 10.1152/ajplung.00189.2015 26702149

[87] Mata M Sarrion I Milian L Juan G Ramon M Naufal D PGC-1alpha induction in pulmonary arterial hypertension. *Oxid Med Cell Longev.* (2012) 2012:236572. 10.1155/2012/236572 22973467 PMC3437671

[88] Ryan JJ Marsboom G Fang YH Toth PT Morrow E Luo N PGC1alpha-mediated mitofusin-2 deficiency in female rats and humans with pulmonary arterial hypertension. *Am J Respir Crit Care Med.* (2013) 187:865–78. 10.1164/rccm.201209-1687OC 23449689 PMC3707374

[89] Ghatnekar A Trojanowska M. GATA-6 is a novel transcriptional repressor of the human *Tenascin-C* gene expression in fibroblasts. *Biochim Biophys Acta.* (2008) 1779:145–51. 10.1016/j.bbagrm.2007.11.012 18177748 PMC2295212

[90] Bourgeois A Lambert C Habbout K Ranchoux B Paquet-Marceau S Trinh I FOXM1 promotes pulmonary artery smooth muscle cell expansion in pulmonary arterial hypertension. *J Mol Med.* (2018) 96:223–35. 10.1007/s00109-017-1619-0 29290032

[91] Li Z You Q Zhang X. Small-molecule modulators of the hypoxia-inducible factor pathway: development and therapeutic applications. *J Med Chem.* (2019) 62:5725–49. 10.1021/acs.jmedchem.8b01596 30682255

[92] Raghavan A Zhou G Zhou Q Ibe JC Ramchandran R Yang Q Hypoxia-induced pulmonary arterial smooth muscle cell proliferation is controlled by forkhead box M1. *Am J Respir Cell Mol Biol.* (2012) 46:431–6. 10.1165/rcmb.2011-0128OC 22033266 PMC3359951

[93] Savai R Al-Tamari HM Sedding D Kojonazarov B Muecke C Teske R Pro-proliferative and inflammatory signaling converge on FoxO1 transcription factor in pulmonary hypertension. *Nat Med.* (2014) 20:1289–300. 10.1038/nm.3695 25344740

[94] Oikawa T. ETS transcription factors: possible targets for cancer therapy. *Cancer Sci.* (2004) 95:626–33. 10.1111/j.1349-7006.2004.tb03320.x 15298723 PMC11159856

[95] Patel M Predescu D Tandon R Bardita C Pogoriler J Bhorade S A novel p38 mitogen-activated protein kinase/Elk-1 transcription factor-dependent molecular mechanism underlying abnormal endothelial cell proliferation in plexogenic pulmonary arterial hypertension. *J Biol Chem.* (2013) 288:25701–16. 10.1074/jbc.M113.502674 23893408 PMC3764778

[96] Austin ED Menon S Hemnes AR Robinson LR Talati M Fox KL Idiopathic and heritable PAH perturb common molecular pathways, correlated with increased MSX1 expression. *Pulm Circ.* (2011) 1:389–98. 10.4103/2045-8932.87308 22140629 PMC3224431

[97] Lengner CJ Welstead GG Jaenisch R. The pluripotency regulator Oct4: a role in somatic stem cells? *Cell Cycle.* (2008) 7:725–8. 10.4161/cc.7.6.5573 18239456

[98] Firth AL Yao W Remillard CV Ogawa A Yuan JX. Upregulation of Oct-4 isoforms in pulmonary artery smooth muscle cells from patients with pulmonary arterial hypertension. *Am J Physiol Lung Cell Mol Physiol.* (2010) 298:L548–57. 10.1152/ajplung.00314.2009 20139178 PMC2853339

[99] Covello KL Kehler J Yu H Gordan JD Arsham AM Hu CJ HIF-2alpha regulates Oct-4: effects of hypoxia on stem cell function, embryonic development, and tumor growth. *Genes Dev.* (2006) 20:557–70. 10.1101/gad.1399906 16510872 PMC1410808

[100] Eul B Rose F Krick S Savai R Goyal P Klepetko W Impact of HIF-1alpha and HIF-2alpha on proliferation and migration of human pulmonary artery fibroblasts in hypoxia. *FASEB J.* (2006) 20:163–5. 10.1096/fj.05-4104fje 16263938

[101] Wu Y Wharton J Walters R Vasilaki E Aman J Zhao L The pathophysiological role of novel pulmonary arterial hypertension gene *SOX17*. *Eur Respir J.* (2021) 58:2004172. 10.1183/13993003.04172-2020 33632800

[102] Sun X Sun BL Babicheva A Vanderpool R Oita RC Casanova N Direct extracellular NAMPT involvement in pulmonary hypertension and vascular remodeling. transcriptional regulation by SOX and HIF-2alpha. *Am J Respir Cell Mol Biol.* (2020) 63:92–103. 10.1165/rcmb.2019-0164OC 32142369 PMC7328254

[103] Graf S Haimel M Bleda M Hadinnapola C Southgate L Li W Identification of rare sequence variation underlying heritable pulmonary arterial hypertension. *Nat Commun.* (2018) 9:1416. 10.1038/s41467-018-03672-4 29650961 PMC5897357

[104] Zhu N Welch CL Wang J Allen PM Gonzaga-Jauregui C Ma L Rare variants in *SOX17* are associated with pulmonary arterial hypertension with congenital heart disease. *Genome Med.* (2018) 10:56. 10.1186/s13073-018-0566-x 30029678 PMC6054746

[105] Rhodes CJ Batai K Bleda M Haimel M Southgate L Germain M Genetic determinants of risk in pulmonary arterial hypertension: international genome-wide association studies and meta-analysis. *Lancet Respir Med.* (2019) 7:227–38. 10.1016/S2213-2600(18)30409-0 30527956 PMC6391516

[106] Li D Shao NY Moonen JR Zhao Z Shi M Otsuki S ALDH1A3 coordinates metabolism with gene regulation in pulmonary arterial hypertension. *Circulation.* (2021) 143:2074–90. 10.1161/CIRCULATIONAHA.120.048845 33764154 PMC8289565

[107] Kim J Hwangbo C Hu X Kang Y Papangeli I Mehrotra D Restoration of impaired endothelial myocyte enhancer factor 2 function rescues pulmonary arterial hypertension. *Circulation.* (2015) 131:190–9. 10.1161/CIRCULATIONAHA.114.013339 25336633 PMC4293354

[108] Muller MR Rao A. NFAT, immunity and cancer: a transcription factor comes of age. *Nat Rev Immunol.* (2010) 10:645–56. 10.1038/nri2818 20725108

[109] He RL Wu ZJ Liu XR Gui LX Wang RX Lin MJ. Calcineurin/NFAT signaling modulates pulmonary artery smooth muscle cell proliferation, migration and apoptosis in monocrotaline-induced pulmonary arterial hypertension rats. *Cell Physiol Biochem.* (2018) 49:172–89. 10.1159/000492852 30134231

[110] Meloche J Pflieger A Vaillancourt M Paulin R Potus F Zervopoulos S Role for DNA damage signaling in pulmonary arterial hypertension. *Circulation.* (2014) 129:786–97. 10.1161/CIRCULATIONAHA.113.006167 24270264

[111] Liang OD So EY Egan PC Goldberg LR Aliotta JM Wu KQ Endothelial to haematopoietic transition contributes to pulmonary arterial hypertension. *Cardiovasc Res.* (2017) 113:1560–73. 10.1093/cvr/cvx161 29016733 PMC5852529

[112] Wigle DA Thompson KE Yablonsky S Zaidi SH Coulber C Jones PL AML1-like transcription factor induces serine elastase activity in ovine pulmonary artery smooth muscle cells. *Circ Res.* (1998) 83:252–63. 10.1161/01.res.83.3.252 9710117

[113] Lin ME Chen T Leaf EM Speer MY Giachelli CM. *Runx2* expression in smooth muscle cells is required for arterial medial calcification in mice. *Am J Pathol.* (2015) 185:1958–69. 10.1016/j.ajpath.2015.03.020 25987250 PMC4484217

[114] Lin ME Chen TM Wallingford MC Nguyen NB Yamada S Sawangmake C *Runx2* deletion in smooth muscle cells inhibits vascular osteochondrogenesis and calcification but not atherosclerotic lesion formation. *Cardiovasc Res.* (2016) 112:606–16. 10.1093/cvr/cvw205 27671804 PMC5079276

[115] Sun Y Byon CH Yuan K Chen J Mao X Heath JM Smooth muscle cell-specific *Runx2* deficiency inhibits vascular calcification. *Circ Res.* (2012) 111:543–52. 10.1161/CIRCRESAHA.112.267237 22773442 PMC3678289

[116] Ruffenach G Chabot S Tanguay VF Courboulin A Boucherat O Potus F Role for runt-related transcription factor 2 in proliferative and calcified vascular lesions in pulmonary arterial hypertension. *Am J Respir Crit Care Med.* (2016) 194:1273–85. 10.1164/rccm.201512-2380OC 27149112

[117] Toufektchan E Toledo F. The guardian of the genome revisited: p53 downregulates genes required for telomere maintenance, DNA repair, and centromere structure. *Cancers.* (2018) 10:135. 10.3390/cancers10050135 29734785 PMC5977108

[118] Hafner A Bulyk ML Jambhekar A Lahav G. The multiple mechanisms that regulate p53 activity and cell fate. *Nat Rev Mol Cell Biol.* (2019) 20:199–210. 10.1038/s41580-019-0110-x 30824861

[119] Fischer M. Census and evaluation of p53 target genes. *Oncogene.* (2017) 36:3943–56. 10.1038/onc.2016.502 28288132 PMC5511239

[120] Kastenhuber ER Lowe SW. Putting p53 in context. *Cell.* (2017) 170:1062–78. 10.1016/j.cell.2017.08.028 28886379 PMC5743327

[121] Chavala SH Kim Y Tudisco L Cicatiello V Milde T Kerur N Retinal angiogenesis suppression through small molecule activation of p53. *J Clin Invest.* (2013) 123:4170–81. 10.1172/JCI67315 24018558 PMC3784529

[122] Gogiraju R Xu X Bochenek ML Steinbrecher JH Lehnart SE Wenzel P Endothelial p53 deletion improves angiogenesis and prevents cardiac fibrosis and heart failure induced by pressure overload in mice. *J Am Heart Assoc.* (2015) 4:e001770. 10.1161/JAHA.115.001770 25713289 PMC4345879

[123] Secchiero P Corallini F Gonelli A Dell’Eva R Vitale M Capitani S Antiangiogenic activity of the MDM2 antagonist nutlin-3. *Circ Res.* (2007) 100:61–9. 10.1161/01.RES.0000253975.76198.ff 17138942

[124] Heo KS Lee H Nigro P Thomas T Le NT Chang E PKCzeta mediates disturbed flow-induced endothelial apoptosis via p53 SUMOylation. *J Cell Biol.* (2011) 193:867–84. 10.1083/jcb.201010051 21624955 PMC3105539

[125] Lee CL Moding EJ Cuneo KC Li Y Sullivan JM Mao L P53 functions in endothelial cells to prevent radiation-induced myocardial injury in mice. *Sci Signal.* (2012) 5:ra52. 10.1126/scisignal.2002918 22827996 PMC3533440

[126] Mizuno S Bogaard HJ Kraskauskas D Alhussaini A Gomez-Arroyo J Voelkel NF P53 gene deficiency promotes hypoxia-induced pulmonary hypertension and vascular remodeling in mice. *Am J Physiol Lung Cell Mol Physiol.* (2011) 300:L753–61. 10.1152/ajplung.00286.2010 21335523

[127] Jacquin S Rincheval V Mignotte B Richard S Humbert M Mercier O Inactivation of p53 is sufficient to induce development of pulmonary hypertension in rats. *PLoS One.* (2015) 10:e0131940. 10.1371/journal.pone.0131940 26121334 PMC4488287

[128] Wakasugi T Shimizu I Yoshida Y Hayashi Y Ikegami R Suda M Role of smooth muscle cell p53 in pulmonary arterial hypertension. *PLoS One.* (2019) 14:e0212889. 10.1371/journal.pone.0212889 30807606 PMC6391010

[129] Mouraret N Marcos E Abid S Gary-Bobo G Saker M Houssaini A Activation of lung p53 by Nutlin-3a prevents and reverses experimental pulmonary hypertension. *Circulation.* (2013) 127:1664–76. 10.1161/CIRCULATIONAHA.113.002434 23513067 PMC3989211

[130] Aggarwal BB. Nuclear factor-kappaB: the enemy within. *Cancer Cell.* (2004) 6:203–8. 10.1016/j.ccr.2004.09.003 15380510

[131] Price LC Caramori G Perros F Meng C Gambaryan N Dorfmuller P Nuclear factor kappa-B is activated in the pulmonary vessels of patients with end-stage idiopathic pulmonary arterial hypertension. *PLoS One.* (2013) 8:e75415. 10.1371/journal.pone.0075415 24124488 PMC3790752

[132] Otsuki S Saito T Taylor S Li D Moonen J-R Marciano DP Monocyte released HERV-K dUTPase engages TLR4 and MCAM causing endothelial mesenchymal transition. *JCI Insight.* (2021) 6:e146416. 10.1172/jci.insight.146416 34185707 PMC8410063

[133] Farkas D Alhussaini AA Kraskauskas D Kraskauskiene V Cool CD Nicolls MR Nuclear factor kappaB inhibition reduces lung vascular lumen obliteration in severe pulmonary hypertension in rats. *Am J Respir Cell Mol Biol.* (2014) 51:413–25. 10.1165/rcmb.2013-0355OC 24684441 PMC4189489

[134] Li L Wei C Kim IK Janssen-Heininger Y Gupta S. Inhibition of nuclear factor-kappaB in the lungs prevents monocrotaline-induced pulmonary hypertension in mice. *Hypertension.* (2014) 63:1260–9. 10.1161/HYPERTENSIONAHA.114.03220 24614212

[135] Bongers EM Duijf PH van Beersum SE Schoots J Van Kampen A Burckhardt A Mutations in the human *TBX4* gene cause small patella syndrome. *Am J Hum Genet.* (2004) 74:1239–48. 10.1086/421331 15106123 PMC1182087

[136] Kerstjens-Frederikse WS Bongers EM Roofthooft MT Leter EM Douwes JM Van Dijk A *TBX4* mutations (small patella syndrome) are associated with childhood-onset pulmonary arterial hypertension. *J Med Genet.* (2013) 50:500–6. 10.1136/jmedgenet-2012-101152 23592887 PMC3717587

[137] Paulin R Courboulin A Meloche J Mainguy V Dumas de la Roque E Saksouk N Signal transducers and activators of transcription-3/pim1 axis plays a critical role in the pathogenesis of human pulmonary arterial hypertension. *Circulation.* (2011) 123:1205–15. 10.1161/CIRCULATIONAHA.110.963314 21382889 PMC3545712

[138] Gairhe S Awad KS Dougherty EJ Ferreyra GA Wang S Yu Z-X Type I interferon activation and endothelial dysfunction in caveolin-1 insufficiency-associated pulmonary arterial hypertension. *Proc Natl Acad Sci U.S.A.* (2021) 118:e2010206118. 10.1073/pnas.2010206118 33836561 PMC7980434

[139] Zabini D Granton E Hu Y Miranda MZ Weichelt U Breuils Bonnet S Loss of SMAD3 promotes vascular remodeling in pulmonary arterial hypertension via MRTF disinhibition. *Am J Respir Crit Care Med.* (2018) 197:244–60. 10.1164/rccm.201702-0386OC 29095649

[140] Reyes-Palomares A Gu M Grubert F Berest I Sa S Kasowski M Remodeling of active endothelial enhancers is associated with aberrant gene-regulatory networks in pulmonary arterial hypertension. *Nat Commun.* (2020) 11:1673. 10.1038/s41467-020-15463-x 32245974 PMC7125148

[141] Garcia-Rivas G Jerjes-Sanchez C Rodriguez D Garcia-Pelaez J Trevino V. A systematic review of genetic mutations in pulmonary arterial hypertension. *BMC Med Genet.* (2017) 18:82. 10.1186/s12881-017-0440-5 28768485 PMC5541665

[142] Aldred MA Morrell NW Guignabert C. New mutations and pathogenesis of pulmonary hypertension: progress and puzzles in disease pathogenesis. *Circ Res.* (2022) 130:1365–81. 10.1161/CIRCRESAHA.122.320084 35482831 PMC9897592

[143] Maston GA Evans SK Green MR. Transcriptional regulatory elements in the human genome. *Annu Rev Genomics Hum Genet.* (2006) 7:29–59. 10.1146/annurev.genom.7.080505.115623 16719718

[144] Aguilar D Oliva B. Topological comparison of methods for predicting transcriptional cooperativity in yeast. *BMC Genomics.* (2008) 9:137. 10.1186/1471-2164-9-137 18366726 PMC2315657

[145] Amoutzias GD Robertson DL Van de Peer Y Oliver SG. Choose your partners: dimerization in eukaryotic transcription factors. *Trends Biochem Sci.* (2008) 33:220–9. 10.1016/j.tibs.2008.02.002 18406148

[146] Wisdom R Johnson RS Moore C. C-Jun regulates cell cycle progression and apoptosis by distinct mechanisms. *EMBO J.* (1999) 18:188–97. 10.1093/emboj/18.1.188 9878062 PMC1171114

[147] Biasin V Chwalek K Wilhelm J Best J Marsh LM Ghanim B Endothelin-1 driven proliferation of pulmonary arterial smooth muscle cells is c-fos dependent. *Int J Biochem Cell Biol.* (2014) 54:137–48. 10.1016/j.biocel.2014.06.020 25016214

[148] Dabral S Tian X Kojonazarov B Savai R Ghofrani HA Weissmann N Notch1 signalling regulates endothelial proliferation and apoptosis in pulmonary arterial hypertension. *Eur Respir J.* (2016) 48:1137–49. 10.1183/13993003.00773-2015 27471204

[149] Miyagawa K Shi M Chen PI Hennigs JK Zhao Z Wang M Smooth muscle contact drives endothelial regeneration by BMPR2-Notch1-mediated metabolic and epigenetic changes. *Circ Res.* (2019) 124:211–24. 10.1161/CIRCRESAHA.118.313374 30582451 PMC6400637

[150] Yu AY Frid MG Shimoda LA Wiener CM Stenmark K Semenza GL. Temporal, spatial, and oxygen-regulated expression of hypoxia-inducible factor-1 in the lung. *Am J Physiol.* (1998) 275:L818–26. 10.1152/ajplung.1998.275.4.L818 9755115

[151] Palmer LA Semenza GL Stoler MH Johns RA. Hypoxia induces type II NOS gene expression in pulmonary artery endothelial cells via HIF-1. *Am J Physiol.* (1998) 274:L212–9. 10.1152/ajplung.1998.274.2.L212 9486205

[152] Jiang BH Rue E Wang GL Roe R Semenza GL. Dimerization, DNA binding, and transactivation properties of hypoxia-inducible factor 1. *J Biol Chem.* (1996) 271:17771–8. 10.1074/jbc.271.30.17771 8663540

[153] Semenza GL. Hypoxia-inducible factors: roles in cardiovascular disease progression, prevention, and treatment. *Cardiovasc Res.* (2022) 10.1093/cvr/cvac089 [Epub ahead of print]. 35687650

[154] Urrutia AA Aragones J. HIF oxygen sensing pathways in lung biology. *Biomedicines.* (2018) 6:68. 10.3390/biomedicines6020068 29882755 PMC6027477

[155] Abe H Semba H Takeda N. The roles of hypoxia signaling in the pathogenesis of cardiovascular diseases. *J Atheroscler Thromb.* (2017) 24:884–94. 10.5551/jat.RV17009 28757538 PMC5587513

[156] Ahmad A Ahmad S Malcolm KC Miller SM Hendry-Hofer T Schaack JB Differential regulation of pulmonary vascular cell growth by hypoxia-inducible transcription factor-1alpha and hypoxia-inducible transcription factor-2alpha. *Am J Respir Cell Mol Biol.* (2013) 49:78–85. 10.1165/rcmb.2012-0107OC 23492195 PMC3727885

[157] Pullamsetti SS Mamazhakypov A Weissmann N Seeger W Savai R. Hypoxia-inducible factor signaling in pulmonary hypertension. *J Clin Invest.* (2020) 130:5638–51. 10.1172/JCI137558 32881714 PMC7598042

[158] Fijalkowska I Xu W Comhair SA Janocha AJ Mavrakis LA Krishnamachary B Hypoxia inducible-factor1alpha regulates the metabolic shift of pulmonary hypertensive endothelial cells. *Am J Pathol.* (2010) 176:1130–8. 10.2353/ajpath.2010.090832 20110409 PMC2832136

[159] Wang J Weigand L Lu W Sylvester JT Semenza GL Shimoda LA. Hypoxia inducible factor 1 mediates hypoxia-induced TRPC expression and elevated intracellular Ca2+ in pulmonary arterial smooth muscle cells. *Circ Res.* (2006) 98:1528–37. 10.1161/01.RES.0000227551.68124.98 16709899

[160] Shimoda LA Fallon M Pisarcik S Wang J Semenza GL. HIF-1 regulates hypoxic induction of NHE1 expression and alkalinization of intracellular pH in pulmonary arterial myocytes. *Am J Physiol Lung Cell Mol Physiol.* (2006) 291:L941–9. 10.1152/ajplung.00528.2005 16766575

[161] Ignarro LJ Buga GM Wood KS Byrns RE Chaudhuri G. Endothelium-derived relaxing factor produced and released from artery and vein is nitric oxide. *Proc Natl Acad Sci U.S.A.* (1987) 84:9265–9. 10.1073/pnas.84.24.9265 2827174 PMC299734

[162] Semenza GL. Perspectives on oxygen sensing. *Cell.* (1999) 98:281–4. 10.1016/s0092-8674(00)81957-1 10458603

[163] Tonelli AR Haserodt S Aytekin M Dweik RA. Nitric oxide deficiency in pulmonary hypertension: pathobiology and implications for therapy. *Pulm Circ.* (2013) 3:20–30. 10.4103/2045-8932.109911 23662172 PMC3641730

[164] Xue C Rengasamy A Le Cras TD Koberna PA Dailey GC Johns RA. Distribution of NOS in normoxic vs. hypoxic rat lung: upregulation of NOS by chronic hypoxia. *Am J Physiol.* (1994) 267:L667–78. 10.1152/ajplung.1994.267.6.L667 7528981

[165] Pullamsetti SS Schermuly RT. Endothelin receptor antagonists in preclinical models of pulmonary hypertension. *Eur J Clin Invest.* (2009) 39 (Suppl. 2):3–13. 10.1111/j.1365-2362.2009.02115.x 19335741

[166] Meloche J Le Guen M Potus F Vinck J Ranchoux B Johnson I MiR-223 reverses experimental pulmonary arterial hypertension. *Am J Physiol Cell Physiol.* (2015) 309:C363–72. 10.1152/ajpcell.00149.2015 26084306

[167] Diebold I Djordjevic T Hess J Gorlach A. Rac-1 promotes pulmonary artery smooth muscle cell proliferation by upregulation of plasminogen activator inhibitor-1: role of NFkappaB-dependent hypoxia-inducible factor-1alpha transcription. *Thromb Haemost.* (2008) 100:1021–8. 19132225

[168] Dabral S Muecke C Valasarajan C Schmoranzer M Wietelmann A Semenza GL A RASSF1A-HIF1alpha loop drives Warburg effect in cancer and pulmonary hypertension. *Nat Commun.* (2019) 10:2130. 10.1038/s41467-019-10044-z 31086178 PMC6513860

[169] Blum JI Bijli KM Murphy TC Kleinhenz JM Hart CM. Time-dependent PPARgamma modulation of HIF-1alpha signaling in hypoxic pulmonary artery smooth muscle cells. *Am J Med Sci.* (2016) 352:71–9. 10.1016/j.amjms.2016.03.019 27432037 PMC5483378

[170] Tang H Babicheva A McDermott KM Gu Y Ayon RJ Song S Endothelial HIF-2alpha contributes to severe pulmonary hypertension due to endothelial-to-mesenchymal transition. *Am J Physiol Lung Cell Mol Physiol.* (2018) 314:L256–75. 10.1152/ajplung.00096.2017 29074488 PMC5866501

[171] Kapitsinou PP Rajendran G Astleford L Michael M Schonfeld MP Fields T The endothelial prolyl-4-hydroxylase domain 2/hypoxia-inducible factor 2 axis regulates pulmonary artery pressure in mice. *Mol Cell Biol.* (2016) 36:1584–94. 10.1128/MCB.01055-15 26976644 PMC4859687

[172] Brusselmans K Compernolle V Tjwa M Wiesener MS Maxwell PH Collen D Heterozygous deficiency of hypoxia-inducible factor-2alpha protects mice against pulmonary hypertension and right ventricular dysfunction during prolonged hypoxia. *J Clin Invest.* (2003) 111:1519–27. 10.1172/JCI15496 12750401 PMC155039

[173] Cowburn AS Crosby A Macias D Branco C Colaco RD Southwood M HIF2alpha-arginase axis is essential for the development of pulmonary hypertension. *Proc Natl Acad Sci U.S.A.* (2016) 113:8801–6. 10.1073/pnas.1602978113 27432976 PMC4978263

[174] Gale DP Harten SK Reid CD Tuddenham EG Maxwell PH. Autosomal dominant erythrocytosis and pulmonary arterial hypertension associated with an activating HIF2 alpha mutation. *Blood.* (2008) 112:919–21. 10.1182/blood-2008-04-153718 18650473

[175] Tan Q Kerestes H Percy MJ Pietrofesa R Chen L Khurana TS Erythrocytosis and pulmonary hypertension in a mouse model of human HIF2A gain of function mutation. *J Biol Chem.* (2013) 288:17134–44. 10.1074/jbc.M112.444059 23640890 PMC3682519

[176] Chan XY Volkova E Eoh J Black R Fang L Gorashi R *HIF2A* gain-of-function mutation modulates the stiffness of smooth muscle cells and compromises vascular mechanics. *iScience.* (2021) 24:102246. 10.1016/j.isci.2021.102246 33796838 PMC7995528

[177] Humbert M Morrell NW Archer SL Stenmark KR MacLean MR Lang IM Cellular and molecular pathobiology of pulmonary arterial hypertension. *J Am Coll Cardiol.* (2004) 43:13–24S. 10.1016/j.jacc.2004.02.029 15194174

[178] Nasim MT Ghouri A Patel B James V Rudarakanchana N Morrell NW Stoichiometric imbalance in the receptor complex contributes to dysfunctional BMPR-II mediated signalling in pulmonary arterial hypertension. *Hum Mol Genet.* (2008) 17:1683–94. 10.1093/hmg/ddn059 18321866

[179] Nohe A Keating E Underhill TM Knaus P Petersen NO. Dynamics and interaction of caveolin-1 isoforms with BMP-receptors. *J Cell Sci.* (2005) 118:643–50. 10.1242/jcs.01402 15657086

[180] Massague J Wotton D. Transcriptional control by the TGF-beta/Smad signaling system. *EMBO J.* (2000) 19:1745–54. 10.1093/emboj/19.8.1745 10775259 PMC302010

[181] Gomez-Puerto MC Iyengar PV Garcia de Vinuesa A Ten Dijke P Sanchez-Duffhues G. Bone morphogenetic protein receptor signal transduction in human disease. *J Pathol.* (2019) 247:9–20. 10.1002/path.5170 30246251 PMC6587955

[182] Harada M Qin Y Takano H Minamino T Zou Y Toko H G-CSF prevents cardiac remodeling after myocardial infarction by activating the Jak-Stat pathway in cardiomyocytes. *Nat Med.* (2005) 11:305–11. 10.1038/nm1199 15723072

[183] Paulin R Meloche J Bonnet S. STAT3 signaling in pulmonary arterial hypertension. *JAKSTAT.* (2012) 1:223–33. 10.4161/jkst.22366 24058777 PMC3670278

[184] Dutzmann J Daniel JM Bauersachs J Hilfiker-Kleiner D Sedding DG. Emerging translational approaches to target STAT3 signalling and its impact on vascular disease. *Cardiovasc Res.* (2015) 106:365–74. 10.1093/cvr/cvv103 25784694 PMC4431663

[185] Bonnet S Rochefort G Sutendra G Archer SL Haromy A Webster L The nuclear factor of activated T cells in pulmonary arterial hypertension can be therapeutically targeted. *Proc Natl Acad Sci U.S.A.* (2007) 104:11418–23. 10.1073/pnas.0610467104 17596340 PMC1903339

[186] Panciera T Azzolin L Cordenonsi M Piccolo S. Mechanobiology of YAP and TAZ in physiology and disease. *Nat Rev Mol Cell Biol.* (2017) 18:758–70. 10.1038/nrm.2017.87 28951564 PMC6192510

[187] Dieffenbach PB Maracle M Tschumperlin DJ Fredenburgh LE. Mechanobiological feedback in pulmonary vascular disease. *Front Physiol.* (2018) 9:951. 10.3389/fphys.2018.00951 30090065 PMC6068271

[188] Bertero T Cottrill KA Lu Y Haeger CM Dieffenbach P Annis S Matrix remodeling promotes pulmonary hypertension through feedback mechanoactivation of the YAP/TAZ-miR-130/301 circuit. *Cell Rep.* (2015) 13:1016–32. 10.1016/j.celrep.2015.09.049 26565914 PMC4644508

[189] Bertero T Oldham WM Cottrill KA Pisano S Vanderpool RR Yu Q Vascular stiffness mechanoactivates YAP/TAZ-dependent glutaminolysis to drive pulmonary hypertension. *J Clin Invest.* (2016) 126:3313–35. 10.1172/JCI86387 27548520 PMC5004943

[190] Dieffenbach PB Haeger CM Coronata AMF Choi KM Varelas X Tschumperlin DJ Arterial stiffness induces remodeling phenotypes in pulmonary artery smooth muscle cells via YAP/TAZ-mediated repression of cyclooxygenase-2. *Am J Physiol Lung Cell Mol Physiol.* (2017) 313:L628–47. 10.1152/ajplung.00173.2017 28642262 PMC5625262

[191] Sutliff RL Kang BY Hart CM. PPARgamma as a potential therapeutic target in pulmonary hypertension. *Ther Adv Respir Dis.* (2010) 4:143–60. 10.1177/1753465809369619 20530063 PMC3978142

[192] Lehrke M Lazar MA. The many faces of PPARgamma. *Cell.* (2005) 123:993–9. 10.1016/j.cell.2005.11.026 16360030

[193] Chandra V Huang P Hamuro Y Raghuram S Wang Y Burris TP Structure of the intact PPAR-gamma-RXR- nuclear receptor complex on DNA. *Nature.* (2008) 456:350–6. 10.1038/nature07413 19043829 PMC2743566

[194] Plutzky J. The PPAR-RXR transcriptional complex in the vasculature: energy in the balance. *Circ Res.* (2011) 108:1002–16. 10.1161/CIRCRESAHA.110.226860 21493923

[195] Khandekar MJ Banks AS Laznik-Bogoslavski D White JP Choi JH Kazak L Noncanonical agonist PPARgamma ligands modulate the response to DNA damage and sensitize cancer cells to cytotoxic chemotherapy. *Proc Natl Acad Sci U.S.A.* (2018) 115:561–6. 10.1073/pnas.1717776115 29295932 PMC5776997

[196] Thenappan T Ormiston ML Ryan JJ Archer SL. Pulmonary arterial hypertension: pathogenesis and clinical management. *BMJ.* (2018) 360:j5492. 10.1136/bmj.j5492 29540357 PMC6889979

[197] Boucly A Savale L Jais X Bauer F Bergot E Bertoletti L Association between Initial treatment strategy and long-term survival in pulmonary arterial hypertension. *Am J Respir Crit Care Med.* (2021) 204:842–54. 10.1164/rccm.202009-3698OC 34185620

[198] Humbert M Lau EM Montani D Jais X Sitbon O Simonneau G. Advances in therapeutic interventions for patients with pulmonary arterial hypertension. *Circulation.* (2014) 130:2189–208. 10.1161/CIRCULATIONAHA.114.006974 25602947

[199] Benza RL Miller DP Barst RJ Badesch DB Frost AE McGoon MD. An evaluation of long-term survival from time of diagnosis in pulmonary arterial hypertension from the REVEAL Registry. *Chest.* (2012) 142:448–56. 10.1378/chest.11-1460 22281797

[200] Hoeper MM Pausch C Grunig E Staehler G Huscher D Pittrow D Temporal trends in pulmonary arterial hypertension: results from the COMPERA registry. *Eur Respir J.* (2022) 59:2102024. 10.1183/13993003.02024-2021 34675047 PMC9160392

[201] Bushweller JH. Targeting transcription factors in cancer - from undruggable to reality. *Nat Rev Cancer.* (2019) 19:611–24. 10.1038/s41568-019-0196-7 31511663 PMC8820243

[202] Henley MJ Koehler AN. Advances in targeting ‘undruggable’ transcription factors with small molecules. *Nat Rev Drug Discov.* (2021) 20:669–88. 10.1038/s41573-021-00199-0 34006959

[203] Defronzo RA Inzucchi S Abdul-Ghani M Nissen SE. Pioglitazone: the forgotten, cost-effective cardioprotective drug for type 2 diabetes. *Diab Vasc Dis Res.* (2019) 16:133–43. 10.1177/1479164118825376 30706731

[204] Sitbon O Gomberg-Maitland M Granton J Lewis MI Mathai SC Rainisio M Clinical trial design and new therapies for pulmonary arterial hypertension. *Eur Respir J.* (2019) 53:1801908. 10.1183/13993003.01908-2018 30545975 PMC6351342

[205] Paulin R Michelakis ED. Addressing complexity in pulmonary hypertension: the FoxO1 case. *Circ Res.* (2015) 116:1732–5. 10.1161/CIRCRESAHA.115.305773 25999418

[206] Calissi G Lam EW Link W. Therapeutic strategies targeting FOXO transcription factors. *Nat Rev Drug Discov.* (2021) 20:21–38. 10.1038/s41573-020-0088-2 33173189

[207] Abud EM Maylor J Undem C Punjabi A Zaiman AL Myers AC Digoxin inhibits development of hypoxic pulmonary hypertension in mice. *Proc Natl Acad Sci U.S.A* (2012) 109:1239–44. 10.1073/pnas.1120385109 22232678 PMC3268303

[208] Macias D Moore S Crosby A Southwood M Du X Tan H Targeting HIF2α-ARNT hetero-dimerisation as a novel therapeutic strategy for pulmonary arterial hypertension. *Eur Respir J.* (2021) 57:1902061. 10.1183/13993003.02061-2019 32972983 PMC7930471

[209] Hu CJ Poth JM Zhang H Flockton A Laux A Kumar S Suppression of HIF2 signalling attenuates the initiation of hypoxia-induced pulmonary hypertension. *Eur Respir J.* (2019) 54:1900378. 10.1183/13993003.00378-2019 31515405 PMC6911916

[210] Ghosh MC Zhang DL Ollivierre WH Noguchi A Springer DA Linehan WM Therapeutic inhibition of HIF-2alpha reverses polycythemia and pulmonary hypertension in murine models of human diseases. *Blood.* (2021) 137:2509–19. 10.1182/blood.2020009138 33512384 PMC8109019

[211] Zheng Q Lu W Yan H Duan X Chen Y Zhang C Established pulmonary hypertension in rats was reversed by a combination of a HIF-2alpha antagonist and a p53 agonist. *Br J Pharmacol.* (2022) 179:1065–81. 10.1111/bph.15696 34599843

[212] Liu H Zhang S Liu Y Ma J Chen W Yin T Knockdown of HSP110 attenuates hypoxia-induced pulmonary hypertension in mice through suppression of YAP/TAZ-TEAD4 pathway. *Respir Res.* (2022) 23:209. 10.1186/s12931-022-02124-4 35986277 PMC9389662

[213] Torres-Capelli M Marsboom G Li QO Tello D Rodriguez FM Alonso T Role Of Hif2alpha oxygen sensing pathway in bronchial epithelial club cell proliferation. *Sci Rep.* (2016) 6:25357. 10.1038/srep25357 27150457 PMC4858655

[214] Pasupneti S Tian W Tu AB Dahms P Granucci E Gandjeva A Endothelial HIF-2alpha as a key endogenous mediator preventing emphysema. *Am J Respir Crit Care Med.* (2020) 202:983–95. 10.1164/rccm.202001-0078OC 32515984 PMC7528783

[215] Klinke A Moller A Pekarova M Ravekes T Friedrichs K Berlin M Protective effects of 10-nitro-oleic acid in a hypoxia-induced murine model of pulmonary hypertension. *Am J Respir Cell Mol Biol.* (2014) 51:155–62. 10.1165/rcmb.2013-0063OC 24521348 PMC4091852

[216] Hansmann G Wagner RA Schellong S Perez VA Urashima T Wang L Pulmonary arterial hypertension is linked to insulin resistance and reversed by peroxisome proliferator-activated receptor-gamma activation. *Circulation.* (2007) 115:1275–84. 10.1161/CIRCULATIONAHA.106.663120 17339547

[217] Behringer A Trappiel M Berghausen EM Ten Freyhaus H Wellnhofer E Odenthal M Pioglitazone alleviates cardiac and vascular remodelling and improves survival in monocrotaline induced pulmonary arterial hypertension. *Naunyn Schmiedebergs Arch Pharmacol.* (2016) 389:369–79. 10.1007/s00210-015-1205-3 26742933

[218] Kozlowska H Baranowska-Kuczko M Schlicker E Kozlowski M Kloza M Malinowska B. Relaxation of human pulmonary arteries by PPARgamma agonists. *Naunyn Schmiedebergs Arch Pharmacol.* (2013) 386:445–53. 10.1007/s00210-013-0846-3 23483194 PMC3622741

[219] Liu Y Tian XY Mao G Fang X Fung ML Shyy JY Peroxisome proliferator-activated receptor-gamma ameliorates pulmonary arterial hypertension by inhibiting 5-hydroxytryptamine 2B receptor. *Hypertension.* (2012) 60:1471–8. 10.1161/HYPERTENSIONAHA.112.198887 23108648

[220] Kang BY Park KK Kleinhenz JM Murphy TC Green DE Bijli KM Peroxisome proliferator-activated receptor gamma and microRNA 98 in hypoxia-induced endothelin-1 signaling. *Am J Respir Cell Mol Biol.* (2016) 54:136–46. 10.1165/rcmb.2014-0337OC 26098770 PMC4742924

[221] Zauli G Celeghini C Melloni E Voltan R Ongari M Tiribelli M The sorafenib plus nutlin-3 combination promotes synergistic cytotoxicity in acute myeloid leukemic cells irrespectively of FLT3 and p53 status. *Haematologica.* (2012) 97:1722–30. 10.3324/haematol.2012.062083 22689683 PMC3487447

[222] Mascarenhas J Lu M Kosiorek H Virtgaym E Xia L Sandy L Oral idasanutlin in patients with polycythemia vera. *Blood.* (2019) 134:525–33. 10.1182/blood.2018893545 31167802 PMC6688433

[223] Italiano A Miller WH Jr Blay JY Gietema JA Bang YJ Mileshkin LR Phase I study of daily and weekly regimens of the orally administered MDM2 antagonist idasanutlin in patients with advanced tumors. *Invest New Drugs.* (2021) 39:1587–97. 10.1007/s10637-021-01141-2 34180037 PMC8541972

[224] Gamen E Seeger W Pullamsetti SS. The emerging role of epigenetics in pulmonary hypertension. *Eur Respir J.* (2016) 48:903–17. 10.1183/13993003.01714-2015 27492834

[225] Ho L Hossen N Nguyen T Vo A Ahsan F. Epigenetic mechanisms as emerging therapeutic targets and microfluidic chips application in pulmonary arterial hypertension. *Biomedicines.* (2022) 10:170. 10.3390/biomedicines10010170 35052850 PMC8773438

[226] Rhodes CJ Im H Cao A Hennigs JK Wang L Sa S RNA sequencing analysis detection of a novel pathway of endothelial dysfunction in pulmonary arterial hypertension. *Am J Respir Crit Care Med.* (2015) 192:356–66. 10.1164/rccm.201408-1528OC 26030479 PMC4584250

[227] Ainscough AJ Smith TJ Haensel M Rhodes CJ Fellows A Whitwell HJ An organ-on-chip model of pulmonary arterial hypertension identifies a BMPR2-SOX17-prostacyclin signalling axis. *Commun Biol.* (2022) 5:1192. 10.1038/s42003-022-04169-z 36344664 PMC9640600

[228] Spiekerkoetter E Goncharova EA Guignabert C Stenmark K Kwapiszewska G Rabinovitch M Hot topics in the mechanisms of pulmonary arterial hypertension disease: cancer-like pathobiology, the role of the adventitia, systemic involvement, and right ventricular failure. *Pulm Circ.* (2019) 9:2045894019889775. 10.1177/2045894019889775 31798835 PMC6868582

[229] Hennigs JK Matuszcak C Trepel M Korbelin J. Vascular endothelial cells: heterogeneity and targeting approaches. *Cells.* (2021) 10:2712. 10.3390/cells10102712 34685692 PMC8534745

[230] Korbelin J Sieber T Michelfelder S Lunding L Spies E Hunger A Pulmonary targeting of adeno-associated viral vectors by next-generation sequencing-guided screening of random capsid displayed peptide libraries. *Mol Ther.* (2016) 24:1050–61. 10.1038/mt.2016.62 27018516 PMC4923327

[231] Remes A Korbelin J Arnold C Rowedder C Heckmann M Mairbaurl H Adeno-associated virus-mediated gene transfer of inducible nitric oxide synthase to an animal model of pulmonary hypertension. *Hum Gene Ther.* (2022) 33:959–67. 10.1089/hum.2021.230 35850528

[232] Hu H Miao YR Jia LH Yu QY Zhang Q Guo AY. AnimalTFDB 3.0: a comprehensive resource for annotation and prediction of animal transcription factors. *Nucleic Acids Res.* (2019) 47:D33–8. 10.1093/nar/gky822 30204897 PMC6323978

[233] Sysol JR Natarajan V Machado RF. PDGF induces SphK1 expression via Egr-1 to promote pulmonary artery smooth muscle cell proliferation. *Am J Physiol Cell Physiol.* (2016) 310:C983–92. 10.1152/ajpcell.00059.2016 27099350 PMC4935200

[234] Shatat MA Tian H Zhang R Tandon G Hale A Fritz JS Endothelial *Kruppel*-like factor 4 modulates pulmonary arterial hypertension. *Am J Respir Cell Mol Biol.* (2014) 50:647–53. 10.1165/rcmb.2013-0135OC 24156273 PMC4068930

[235] Li Y Wu F Tan Q Guo M Ma P Wang X The multifaceted roles of FOXM1 in pulmonary disease. *Cell Commun Signal.* (2019) 17:35. 10.1186/s12964-019-0347-1 30992007 PMC6469073

[236] Chen R Yan J Liu P Wang Z Wang C Zhong W The role of nuclear factor of activated T cells in pulmonary arterial hypertension. *Cell Cycle.* (2017) 16:508–14. 10.1080/15384101.2017.1281485 28103134 PMC5384583

